# Dealing with AFLP genotyping errors to reveal genetic structure in *Plukenetia volubilis* (Euphorbiaceae) in the Peruvian Amazon

**DOI:** 10.1371/journal.pone.0184259

**Published:** 2017-09-14

**Authors:** Jakub Vašek, Petra Hlásná Čepková, Iva Viehmannová, Martin Ocelák, Danter Cachique Huansi, Pavel Vejl

**Affiliations:** 1 Department of Genetics and Breeding, Faculty of Agrobiology, Food and Natural Resources, Czech University of Life Sciences Prague, Kamýcká, Prague, Czech Republic; 2 Gene Bank, Division of Crop Genetics and Breeding, Crop Research Institute, Drnovská, Prague, Czech Republic; 3 Department of Crop Sciences and Agroforestry, Faculty of Tropical AgriSciences, Czech University of Life Sciences Prague, Kamýcká, Prague, Czech Republic; 4 Peruvian Amazon Research Institute, Tarapoto, Peru; University of Innsbruck, AUSTRIA

## Abstract

An analysis of the population structure and genetic diversity for any organism often depends on one or more molecular marker techniques. Nonetheless, these techniques are not absolutely reliable because of various sources of errors arising during the genotyping process. Thus, a complex analysis of genotyping error was carried out with the AFLP method in 169 samples of the oil seed plant *Plukenetia volubilis* L. from small isolated subpopulations in the Peruvian Amazon. Samples were collected in nine localities from the region of San Martin. Analysis was done in eight datasets with a genotyping error from 0 to 5%. Using eleven primer combinations, 102 to 275 markers were obtained according to the dataset. It was found that it is only possible to obtain the most reliable and robust results through a multiple-level filtering process. Genotyping error and software set up influence both the estimation of population structure and genetic diversity, where in our case population number (K) varied between 2–9 depending on the dataset and statistical method used. Surprisingly, discrepancies in K number were caused more by statistical approaches than by genotyping errors themselves. However, for estimation of genetic diversity, the degree of genotyping error was critical because descriptive parameters (He, F_ST_, PLP 5%) varied substantially (by at least 25%). Due to low gene flow, *P*. *volubilis* mostly consists of small isolated subpopulations (Φ_PT_ = 0.252–0.323) with some degree of admixture given by socio-economic connectivity among the sites; a direct link between the genetic and geographic distances was not confirmed. The study illustrates the successful application of AFLP to infer genetic structure in non-model plants.

## Introduction

*Plukenetia volubilis* L. (sacha inchi) is a climbing, perennial, oleaginous plant of the Euphorbiaceae family which grows in the tropical jungles of the Americas at altitudes between 200 and 1500 m [1]. This species was found in the Lesser Antilles, Surinam, and along the northern and western edge of the Amazon basin in Peru in disturbed areas or forest edge of moist or wet lowland forest [2]. The seeds were used by the native Mochica-Chimú tribe from Pre-Incas times, as suggested through ceramics found in graves and this plant has a long tradition as a food source for the local tribal groups of the region [3]. According to Guttieréz et al. [4] sacha inchi seeds are rich in oil (41.4%) and protein (24.7%), and some minerals are present. Moreover, sacha inchi oil contains many bioactive chemical compounds, which could be beneficial for human health, such as ω-3 fatty acid, phytosterols, tocopherols, and carotenoids [5–7]. *P*. *volubilis* is allogamous and pollination occurs mainly by wind (up to 90%) (pers. com.). In the wild the seeds may be distributed by rodents and other wild animals. Until recently, *P*. *volubilis* was not intentionally bred and the breeding is mainly based on selection for seed size. Present breeding programs are aimed at improving agriculturally important traits, such as yield, because of the potential to utilize the seed oil in different sectors of industry. However, the breeding program for *P*. *volubilis* is still in the initial stages and characterizing its genetic variability is a necessary step towards improving and accelerating the breeding process.

Here, we attempted to obtain high quality data for the estimation of the genetic diversity and population structure of *P*. *volubilis*. The reliability and consistency of results in genetic studies and the quality of datasets used for statistical evaluation have been discussed, particularly regarding the amplified fragment length polymorphism (AFLP) method and the generation of a high number of dominant markers [8–10]. AFLP is a very useful tool for population analyses of species without prior knowledge of their genome [11–14], although a wide range of other applications is possible [15]. Moreover, AFLP is considered to be the most reliable method among the dominant marker producing techniques compared to the RAPD and ISSR methods. Nonetheless, it still suffers from several weaknesses, such as relatively high genotyping error [10] or size homoplasy [16] (more detailed info can be found in reviews by [17] or [18]).

Due to the many steps during laboratory work and subsequent data analysis, there are multiple sources of genotyping error that can have a great impact on the results of population studies [19]. Over the last decade, several strategies have been suggested to mitigate this problem, mostly aimed at the automatization of raw signal processing, data filtering, and allele calling [19–22]. Only a few authors have studied the effect of genotyping error on the estimation of population structure [10] or genetic diversity [23]. For example, Zhang and Hare [10] showed that, although the typical reported error is less than or around 5%, one cannot be sure about the true population structure. These authors obtained different estimations of the number of populations from the same data with error rates of < 2% and > 4% against an error rate within the interval of ≥ 2% and < 4%. Moreover, the estimation of various population parameters is not affected only by the genotyping error, but also by the software used and its setup. This was clearly demonstrated by Arrigo et al. [21], who showed significant discrepancies between the estimation of heterozygosity, the percentage of polymorphic loci and other descriptive statistics of the same data as a result of using different software packages.

In the present study, the general steps leading to improving the precision of AFLP analysis are discussed. Firstly, (i) the minimization of genotyping error at multiple levels. Secondly, (ii) different error rate datasets were created, with the aim of analyzing their impact on the estimation of population structure and genetic diversity. Then, (iii) the population structure of *P*. *volubilis* was assessed, and finally (iv) the relationship between the spatial and genetic structure and the landscape setting was explored.

## Materials and methods

### Ethics statement

This study was not carried out in national parks or other protected areas of land or sea or on private land. Sampling was conducted under the auspice of Danter Cachique Huansi (co-author) from the Peruvian Amazon Research Institute (PARI), Tarapoto, Peru as a responsible person for *Plukenetia volubilis* cultivation and breeding in PARI.

Specific permission was not required for this location and activity. We confirm that the field study did not involve endangered or protected species.

### Sample collection

**Fig 1:**
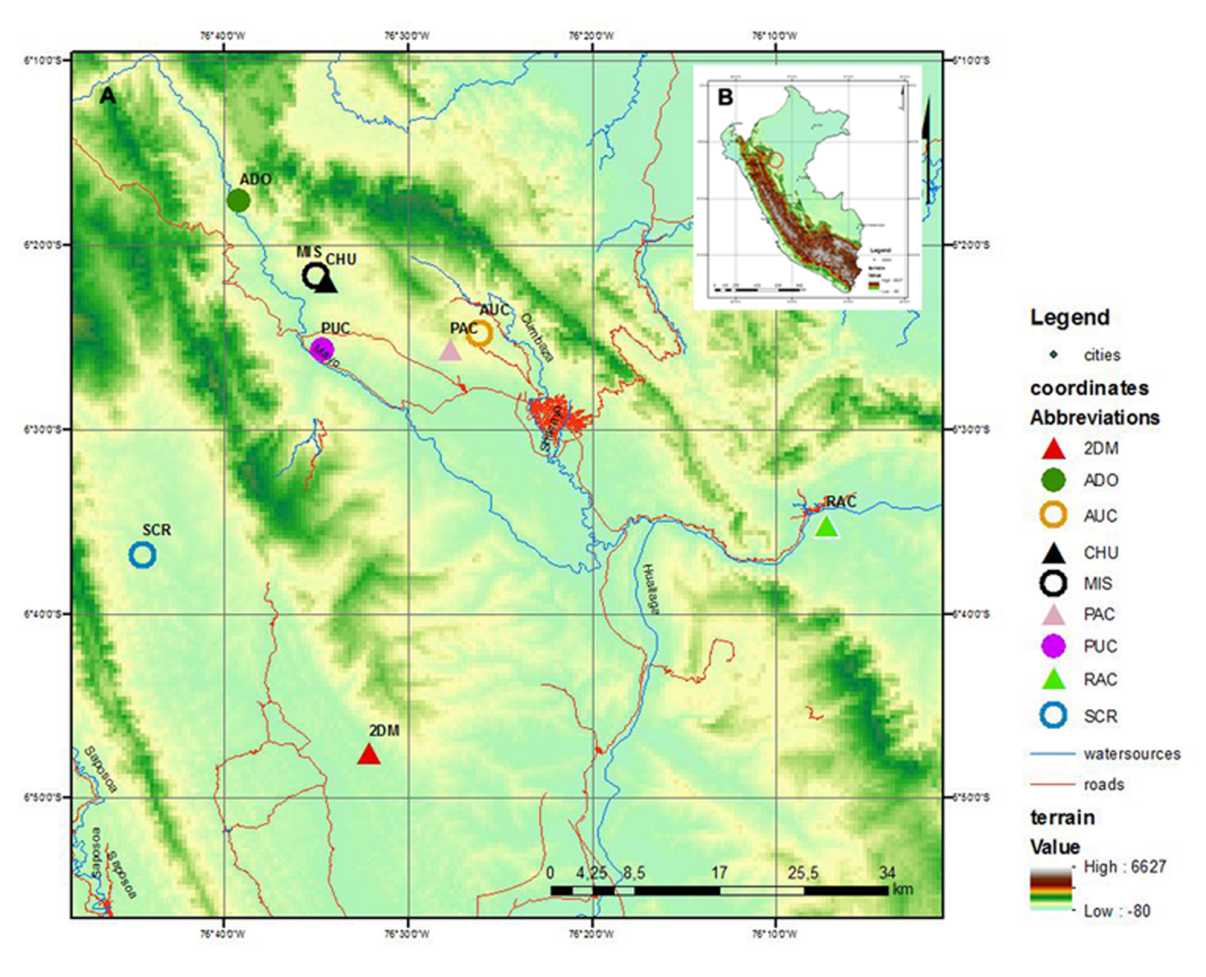
Maps showing sampling locations of *P*. *volubilis* in the Peruvian Amazon. Inset: Map A shows the San Martin region with the positions of *P*. *volubilis* sampling in detail. Map B shows the localization of San Martin within the whole of Peru.

**Fig 2:**
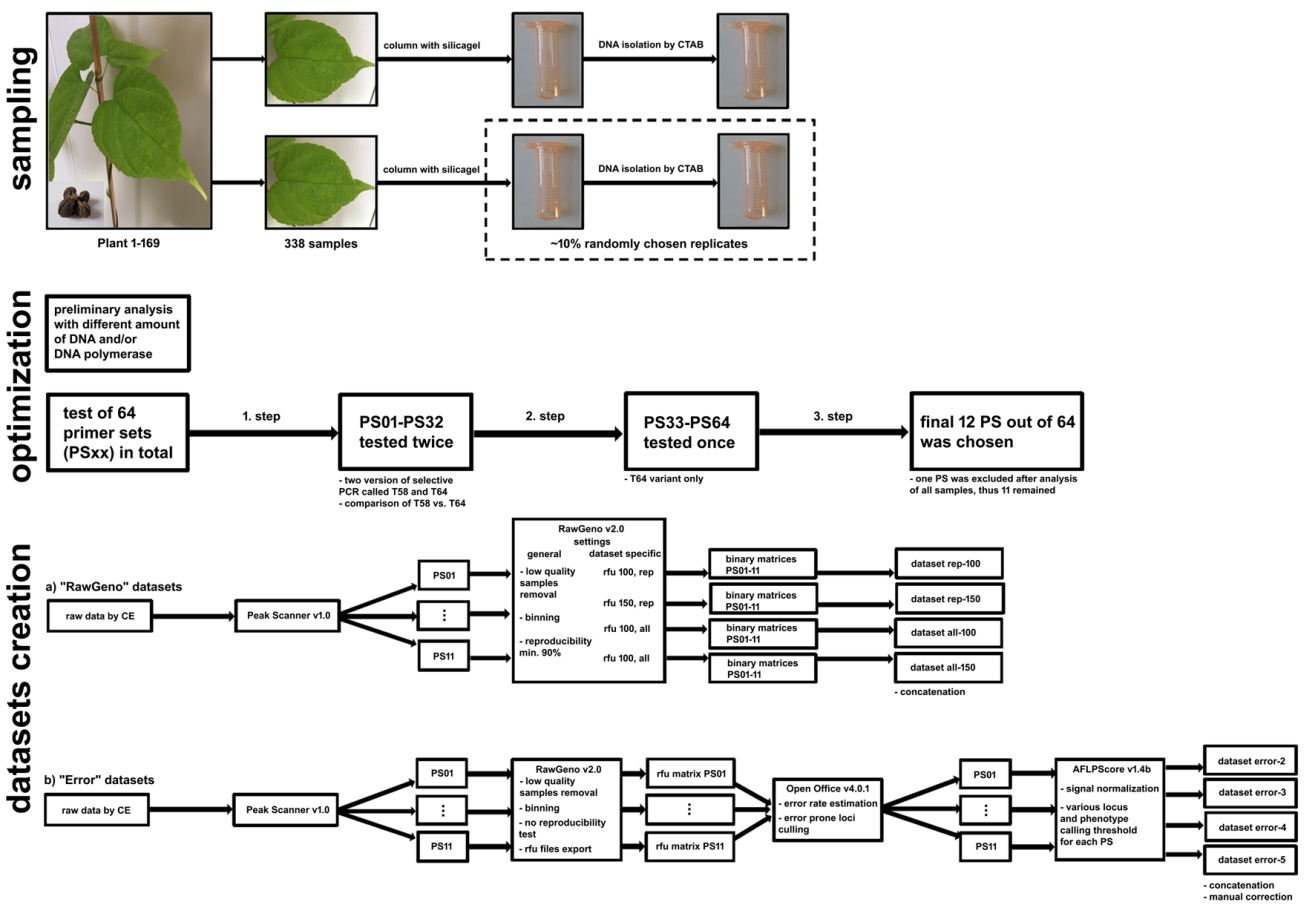
Workflow.

Sampling of fresh leaves suitable for DNA isolation was performed on a panel of 169 *P*. *volubilis* plants collected from nine different localities in the San Martin Region in the Peruvian Amazon (Fig 1, S1 Table). Due to the long-term cultivation of sacha inchi from the times of Pre-Incas, the localities of natural occurrence have not been observed. Our locations correspond to rural fields on which sacha inchi is cultivated by local farmers. The plants are neither bred nor commercially cultivated, except for plants from the localities of Pucallpa (PUC) and Pacchilla (PAC). Depending on accessibility, approximately 20 individuals per population were collected except the Ramón Castillo population (RAC). Within each population, individuals were collected along linear transects at least 15 m apart if possible. In the case of Ramón Castillo (RAC), seven plants were scattered in an abandoned field. All plant samples were collected in replicates (Fig 2). The obtained material was stored immediately in polypropylene tubes with silica gel used as a desiccant. In this way, it was possible to store the plant material for a longer period of time (several weeks) between collection and DNA isolation.

Genomic DNA was isolated by the standard CTAB protocol [24]. Concentration and purity were measured using an S-111107AW nanophotometer (Implen, Germany) and DNA was then diluted to a concentration of 50 ng/μl. The high molecular weight of the DNA was verified by electrophoretic separation on a 1% (w/v) agarose gel.

### AFLP protocol, optimization and raw data generation

Individual steps, temperature profiles and the reaction mixture composition were made according to the original AFLP protocol [25] with recommendations suggested by Meudt and Clarke [15] and Hasbún et al. [26] (See S1 AFLP Protocol). Briefly, DNA restriction by EcoRI and MseI enzymes together with adapter ligation was performed in a single step. The reaction mixture was diluted 1:1 in low TE after 3-hour incubation at 37°C and first (preselective) PCR with annealing temperature at 56°C followed. Amplicons of preselective PCR were diluted 1:9 in low TE and 2.5 μl of PCR products were added with other components to the reaction mixture of the second (selective) PCR. Two variants of the selective PCR (called T58 and T64 according to final annealing temperature) differing in temperature profiles were tested as part of the optimization process (Fig 2). Full protocol with reaction mixtures compound and PCR temperature profiles is available in the Supporting Information, together with details about the number of obtained bins for each primer set under given conditions (S2A–S2C Table).

**Fig 3:**
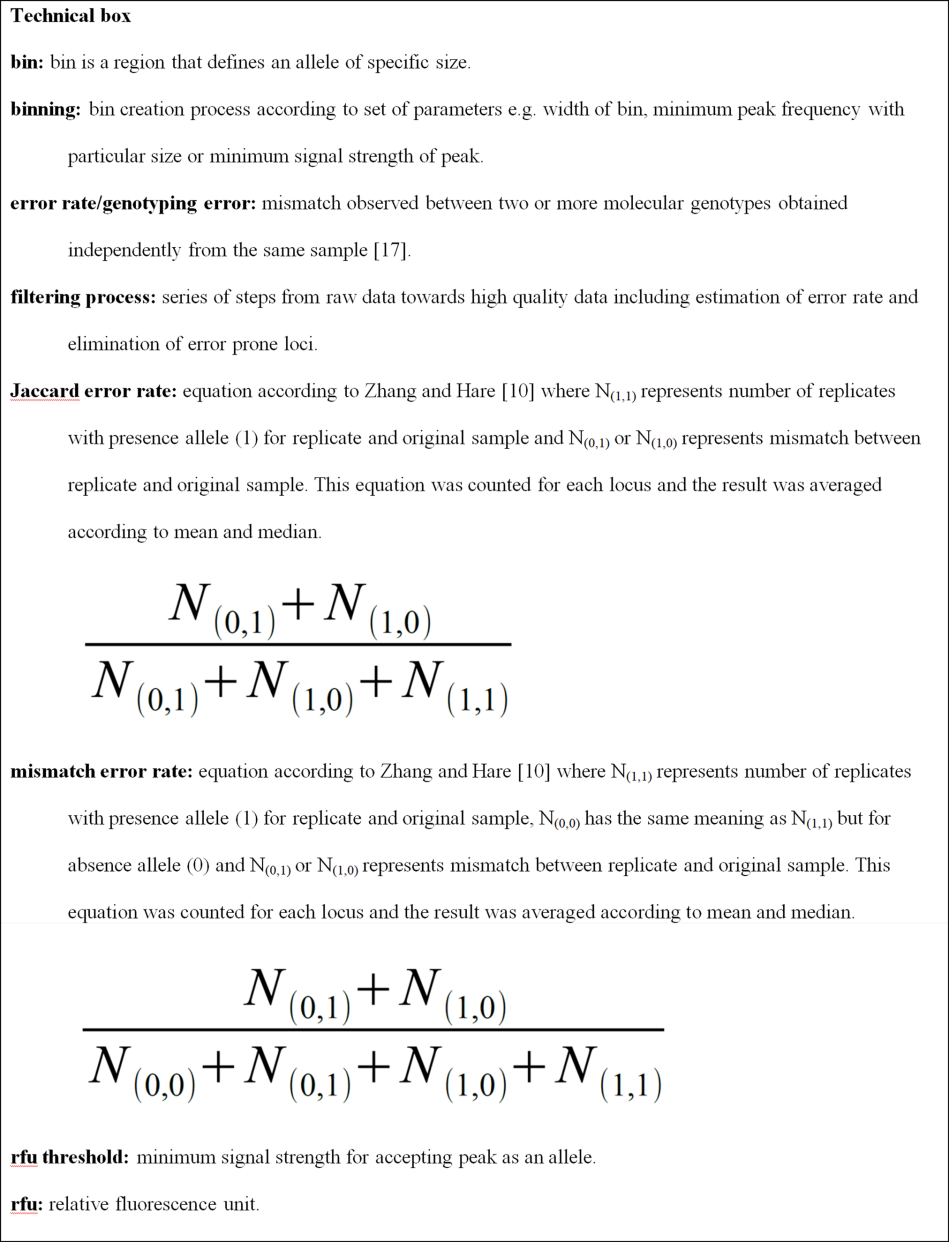
Technical box.

In total, 64 primer sets (PS) were tested on 12 samples originating from all studied locations. Finally, 12 primer sets were chosen for the analysis of all samples, but due to poor signal quality one PS was excluded before data filtering, thus 11 PS remained (Fig 2). The final number of bins of the 32 primer sets was used as the main criterion for comparison of T58 and T64 variant of the selective PCR. Differences in the final number of bins were compared by two-way ANOVA in STATISTICA v12 software (StatSoft Inc.). Categorical factors were temperature profile (T58 vs. T64) and rfu threshold (100 vs. 150 rfu; referred to as rfu_100_ and rfu_150_) (Fig 3). Exploratory data analysis (EDA) was performed before the ANOVA in order to verify the assumptions for the use of this method. The verification of the normal distribution was conducted on the basis of graphical analysis (histograms, box plots) and the Shapiro-Wilk W test. Simultaneously, the homoscedasticity of variance was verified by Levene’s omnibus test. T64 was chosen as a suitable temperature for selective amplification.

Prior to capillary electrophoresis separation, four differently labelled products of selective PCR were poolplexed in the ratio 0.8 (6FAM): 0.9 (VIC): 1.4 (NED): 1.9 (PET) and then diluted with water 1:1. One microliter of pooled amplicons was mixed with 12 μl of Hi-Di formamide (Life Technologies) and 0.2 μl of size standard GeneScan LIZ600® (Life Technologies). PCR products were loaded on an ABI PRISM 310 genetic analyzer (Life Technologies) and separated in a 36-cm long capillary with POP-4^TM^ polymer. Separation conditions were an injection time of 5 s at 10 kV and a running voltage of 13 kV.

For greater clarity and easier data manipulation in downstream analyses, the names of *.fsa files were shortened in the program Ant Renamer v2.10.0 created by A. Potten (http://www.antp.be/software/renamer). The freeware program Peak Scanner^TM^ v1.0 (Life Technologies) was used for signal detection and sizing with set parameters: light peak smoothing, range 50–600 bp, and minimum peak heights 50 rfu. The other parameters remained as the default settings. The limit of 50 rfu was based on testing of instrumental noise in 24 samples containing only pure water both for a new capillary (<100 runs) and a used capillary (>600 runs). The number of noisy peaks was counted for three minimum peak thresholds, i.e. 50, 100 and 150 rfu.

### Error rate analysis

Sixteen randomly selected samples were used as replicates (~10% of analyzed “original” samples) for the reproducibility test. The only condition was that at least one sample per locality must be included. All replicates were treated in the same way as the original samples, i.e. independent DNA isolation, PCR amplification and capillary electrophoresis separation were done (Fig 2).

Detection of size homoplasy was done by a linear regression model according to Vekemans et al. [27] together with size homoplasy decomposition where different criteria for minimum fragment size were tested.

Genotyping quality is also affected by the software and selection criteria settings used [28]. For this reason, eight datasets were created in order to evaluate the influence of at least some of these parameters. RawGeno v2.0 [21] with different settings was used to generate the first four datasets (“RawGeno” datasets) and the remaining datasets (“Error” datasets) were created by combining procedures in RawGeno v2.0 and AFLPScore v1.4b software [20].

#### a) RawGeno datasets

The raw data of each chosen primer set with peaks of 50 rfu (Fig 3) and higher were imported into RawGeno v2.0, analyzed and then concatenated into a single binary matrix. In order to compare the influence of some parameters, four datasets were created (all-100, all-150, rep-100, rep-150) with different minimum peak threshold values (100 or 150 rfu) and keeping (all) or discarding (rep) untested bins according to reproducibility. Prior to binning (Fig 3), a filtering function for low quality samples was used, resulting in the removal of 16 samples, i.e. 153 remained for further analyses. Binning parameters were as follows: maxbin width 1.5 bp, minbin width 1 bp, scoring range 100 bp to the largest fragment size, minimum peak threshold 100 or 150 rfu, low frequency bins 3, reproducibility 90% and keeping (all) or discarding (rep) untested bins. The mismatch error rate (Fig 3) of each combination together with the number of retained loci and summary statistics are shown in Table 1.

**Table 1:** Detailed information on the “RawGeno” datasets showing primer combinations, initial number of bins, number of retained markers, and error rate for each of four datasets.

Primer set		Init. no. of bins	No. of markers		Error rate per set [%]	No. of markers		Error rate per set [%]
			all-100	rep-100		all-150	rep-150	
E02 ACA	M23 CTG	178	40	19	2.50	29	15	1.72
E07 AGG	M19 CAG	148	22	8	0.00	17	7	0.00
E04 ACG	M21 CTA	124	22	13	2.27	18	13	2.78
E01 ACT	M21 CTA	127	27	23	7.41	14	11	0.00
E02 ACA	M21 CTA	126	14	9	0.00	9	7	0.00
E03 AAC	M20 CAT	106	21	15	0.00	17	14	0.00
E04 ACC	M17 CAA	123	30	12	3.33	19	10	2.63
E05 AGC	M19 CAG	193	41	18	2.44	29	15	0.00
E06 AAG	M24 CTT	108	10	5	0.00	5	2	0.00
E07 AGG	M17 CAA	102	19	13	0.00	18	13	0.00
E08 ACG	M22 CTC	118	32	20	3.12	18	13	0.00
	**Total**	1,453	278	155	-	193	120	-
	**Retained markers**		275*	154*		192*	120	
	**Mean**	132	25	14	1.92	18	11	0.65
	**Median**	124	22	13	2.27	18	13	0.00

* Discrepancy in sum of markers against number of retained markers is given by removal of 16 samples and thus several loci during concatenation of 11 datafiles.

#### b) Error datasets

RawGeno v2.0 was used for binning with same the parameters as for the previous four datasets, with the exception that there was no reproducibility testing. The next step was exporting the raw data with rfu values in an AFLPScore compatible format. This was accomplished using an R script published by Lambert et al. [29]. The resulting file was consequently pasted into the Calcul module of Apache OpenOffice v4.0.1. (The Apache Software Foundation). Then, a binary matrix representing the presence/absence of the signal based on the matrix of rfu values was created. This binary matrix was used for the estimation of error rate per locus according to the mismatch error rate [17] and Jaccard error rate [30, 31] (Fig 3); similar ad hoc criteria according to Zhang and Hare [10] were applied where loci with values >18.75% (mismatch) and >31.25% (Jaccard) were removed. The remaining loci of each primer set were exported to AFLPScore v1.4b where choosing the best scoring method was done by testing a broad range of parameters. For the locus selection threshold, the values 50, 100, 150, 200, 250, 300, 400 and 500 rfu were tested. In phenotype calling threshold, the option with absolute values was tested (the same values as in the locus selection threshold) and also those with relative values of 5, 10, 15, 20, 25, 30, 40 and 50%. To assess the effect of the error rate, four datasets were created (error-2, error-3, error-4 and error-5) with different error rates according to the median: 2.35 (the lowest achieved value), 3.13, 4.04 and 5% (Table 2). More information on the selected parameters is presented in the Supporting Information (S3A–S3D Table). The final step was concatenation of all primer combinations into one binary matrix and manual removal of all loci with less than 3 or more than 149 presence peaks, i.e. with a frequency less than 2% or higher than 98% (Fig 2).

**Table 2:** Detailed information on the “Error” dataset showing primer sets, initial number of bins, changes in bin numbers during filtering process, number of retained markers, and error rate for each of the four datasets.

Primer set		Init. no. bins	Primary dataset	Filtered dataset	Final no. loci			
					error-2	error-3	error-4	error-5
E02 ACA	M23 CTG	178	79	54	25	25	29	39
E07 AGG	M19 CAG	148	40	23	17	19	18	23
E04 ACG	M21 CTA	124	50	29	12	9	17	28
E01 ACT	M21 CTA	127	63	22	6	6	9	10
E02 ACA	M21 CTA	126	35	20	6	5	10	17
E03 AAC	M20 CAT	106	35	28	3	3	6	16
E04 ACC	M17 CAA	123	53	33	8	23	33	30
E05 AGC	M19 CAG	193	93	47	30	21	13	30
E06 AAG	M24 CTT	108	35	15	-^#^	-^#^	-^#^	-^#^
E07 AGG	M17 CAA	102	40	27	5	4	12	18
E08 ACG	M22 CTC	118	49	32	12	12	19	31
** **	**Total**	1,453	572	330	124	127	166	242
	**Retained markers**				102*	108*	142*	194*
	**Mean**	132	52	30	12	13	17	24
** **	**Median**	124	49	28	10	11	15	26

* Discrepancy in sum of markers against number of retained markers is given by manual removal of loci with frequency of presence peak below 2% or over 98%.

^#^ This primer set was excluded from the final datasets due to the high error rate and low number of markers.

### Population structure and assignment test

An estimation of the number of populations, the assignment of individuals to a population and their characterization was performed using several mathematically different methods. Four techniques were used: multidimensional scaling (MDS), AMOVA-based K-means clustering, and Bayesian clustering (STRUCTURE—“standard” and “hierarchical” variants). Individual assignment to a population was verified via an assignment test.

#### a. MDS

Distance matrices based on Dice, Jaccard and Simple Matching coefficients were created in DARwin v5.0.158 [32] and used as the input for MDS carried out in STATISTICA v12 (StatSoft Inc.). Only two- and three-dimensional models were tested in the framework of MDS. The quality of fitting the proper model was determined by stress parameter (S6 Table) according to Kruskal [33].

#### b. AMOVA-based K-means clustering

The method was developed by Mermains [34] and implemented in GenoDive v2.0b23 [35]. A distance matrix of individual samples was created according to Smouse and Peakall [36] and the chosen simulated annealing algorithm was set up on 10^6^ Markov chain Monte Carlo (MCMC) iterations with 1000 repeats. The optimal number of clusters (K) was determined by pseudo-F statistics and the Bayesian Information Criterion (BIC).

#### c.Bayesian clustering

Both variants of Bayesian clustering were performed in STRUCTURE v2.3.3 [37] with admixture and correlated allele frequency models [38] and, because AFLP markers are the dominant type of markers, a recessive alleles model [39] was chosen. In the search for a connection between sampling localities and population structure, the LOCPRIOR model [40] was employed. Proper estimation of posterior probability lnP(D) was ensured by 10^6^ MCMC iterations after a burn-in period of 10^5^ iterations and 10 replicate runs, performed for each K value (1–9). The last step comprised 100 replicate runs for the chosen K and only runs with the highest probability were used (see the number of retained runs for each dataset in S1 Fig). This type of STRUCTURE usage is called “standard” analysis.

Evanno et al. [41] pointed out that the parameter lnP(D) used for the estimation of the real number of clusters (K) might not be the best tool and proposed the ad-hoc criterion ΔK, which will detect the uppermost level of the population structure [41, 42] if there is one. For this reason, we also used the repeated procedure of “hierarchical” analysis according to ΔK following Coulon et al. [42] and Lambert et al. [29]. Successive identification of clusters proceeded until the highest probability for K = 1 was reached, the number of individuals within the cluster was too small, or the cluster could not be divided any more (S2 Fig). The criterion for assigning an individual to a subpopulation was based on the highest value of inferred ancestry (at least 0.5). Individuals with a lower value were not assigned to any subpopulation. In every round of the hierarchical analysis, the K value was estimated ranging from 1 to the number of assumed subpopulations with 10 runs per K value. In the case of different numbers of estimated subpopulations in comparison with the result of the standard analysis, 100 replicate runs followed for a chosen K value and only runs with the highest probability were used (S1 Fig). Repeated hierarchical analysis used the same parameter settings as standard analysis.

The calculation of ΔK and averaging lnP(D) values over 10 runs (100 for chosen K) was performed using STRUCTURE HARVESTER v0.6.94 [43] in the case of both “standard” and “hierarchical” analyses. Thus, it was necessary to create a consensus Q matrix using CLUMPP v1.1.2. [44]. Depending on the number of clusters (K), one of three algorithms was used for the calculation of optimal alignment over R replicates. When K ≤ 3, the full-search algorithm was applied, but for K > 3, the greedy algorithm with random input orders and 10^5^ repeats was used. Because of the high number of replicates (mostly ~90 runs per dataset), the large greedy algorithm with 10^6^ random input orders was used for the final runs. The final results were plotted using DISTRUCT v1.1 [45].

#### d. Assignment test

This test was carried out by AFLPOP v1.1 [46]. Prior to our analysis, a series of simulations were conducted for the estimation of the optimal minimum log-likelihood difference (MLD). In order to assure the consistency of the results, each simulation was done in 10 iterations. Based on these simulations, MLD = 1 was chosen. A re-allocation procedure was used for testing and ε = 0.01 was chosen as the zero-replacement value because it is close to the assumed average Bayesian error rate of ε_0.1_ (~0.0084) in the studied datasets (S3A–S3D Table). The probability that a certain individual does not belong to the given candidate subpopulation was set to p < 0.01.

### Genetic diversity

The estimation of allele frequencies using the Bayesian method with a non-uniform prior distribution according to Zhivotovsky et al. [47] with Hardy–Weinberg equilibrium (HWE) assumed was performed in AFLP-SURV v1.0 [27]. These estimations were further used for the calculation of the expected heterozygosity (He) within each subpopulation (S9 Table) following the method of Lynch and Milligan [48] and the detection of size homoplasy [27]. Intrapopulation diversity was measured by other descriptive statistics as the number (#loc_P) and percentage of polymorphic loci (PLP) at the 5% level. F_IS_ value was estimated using I4A program [49] with five different sets of α and β parameters of the prior beta-distribution (α = β = 0.1, 0.5, 1, 2.5 and 5) and posterior distribution for α and β parameters was approximated by 200,000 iterations after burn-in period with 20000 iterations. Final F_IS_ value was counted as a mean of F_IS_ values obtained for each model tested.

The level of interpopulation differentiation was analyzed by analysis of molecular variance (AMOVA) in GenAlEx v6.501 [50] based on the Φ_PT_ parameter because it is more suitable for binary data than F_ST_. Nonetheless, F_ST_ as a routinely used estimator was calculated as well in AFLP-SURV v1.0. The statistical significance of pairwise values Φ_PT_ (F_ST_) for every population was tested by 9999 random permutations.

### Spatial analysis

Testing of the association between genetic and geographical distance was carried out by the Mantel test implemented in GenAlEx v6.501. Input data files were matrices of Φ_PT_ pairwise values (S8A–S8H Table) and the geographical distance matrix based on universal map grid system (UTM) coordinates. The same program was used for spatial autocorrelation analysis according to Smouse and Peakall [36] where seven equidistant classes, each 10 km long, were plotted against r values as a so-called correlogram (S3 Fig). Spatial analysis was furthermore extended by the sPCA method [51] implemented in the Adegenet v2.0.0 package [51] in the R v3.2.1 programming language and environment [52]. The Adegenet package included two permutation based tests used for the detection of global (Gtest) and/or local (Ltest) spatial structures as positive, eventually negative, autocorrelation. The statistical significance of all correlation coefficients of the abovementioned tests was assessed on the basis of 9999 random permutations.

## Results

### AFLP optimization and error rate analysis

#### a) AFLP optimization

Exploratory data analysis (EDA) verified the assumption about normal distribution by Shapiro-Wilk W test (p = 0.08623–0.78834) and homoscedasticity of variance by Levene’s omnibus test (F-value = 2.19; p = 0.14 for the temperature profile and F-value = 0.43; p = 0.51 for the rfu). An appropriate temperature profile was chosen according to the two-way ANOVA results, revealing statistically significant differences both in the temperature profile (F-value = 45.65; p < 0.000001) and the rfu value (F-value = 6.71; p = 0.0107), where the average number of bins was 26.36 for T58 vs. 45.88 for T64 and 39.86 for rfu_100_ vs. 32.37 for rfu_150_.

#### b) Size homoplasy

The analysis was performed for both the concatenated matrices of eight datasets and for individual combinations within every dataset. The results in the form of correlation coefficients r and p-values are listed in S4A and S4B Table. No combination showed a statistically significant negative value r, with the exception of dataset all-100, where a weak negative correlation was detected (r = - 0.1324; p = 0.0282).

#### c) RawGeno and Error datasets

In RawGeno datasets, 25 AFLP markers were obtained per combination for the all-100 dataset, 18 markers for the all-150 dataset, 14 markers for the rep-100 dataset and 11 markers for the rep-150 dataset. The total number of markers, on average, was 19.15, 13.29, 10.67 and 8.26% from the number of bins of raw data.

The “Error” datasets (error-2-5) had 12, 13, 17 and 24 as the average number of markers per combination, which gives a yield of 9.39, 9.61, 12.57 and 18.32% from the initial number of bins. In terms of the error rate, it was found that the error rate per locus ranged from 0 to 75% according to the mismatch error rate (average 17.9%, median 12.5%) and between 0 and 100% according to the Jaccard error rate (average 45.81%, median 40%) for the primary dataset. After filtering, the average error rate was 5.98% (median 6.25%) for the mismatch error rate and 33.08% (median 6.9%) for the Jaccard error rate. It was also found that 191 out of 572 bins (33%) from the primary dataset did not appear in replicated samples and thus were not tested for the error rate.

### Population structure

**Fig 4:**
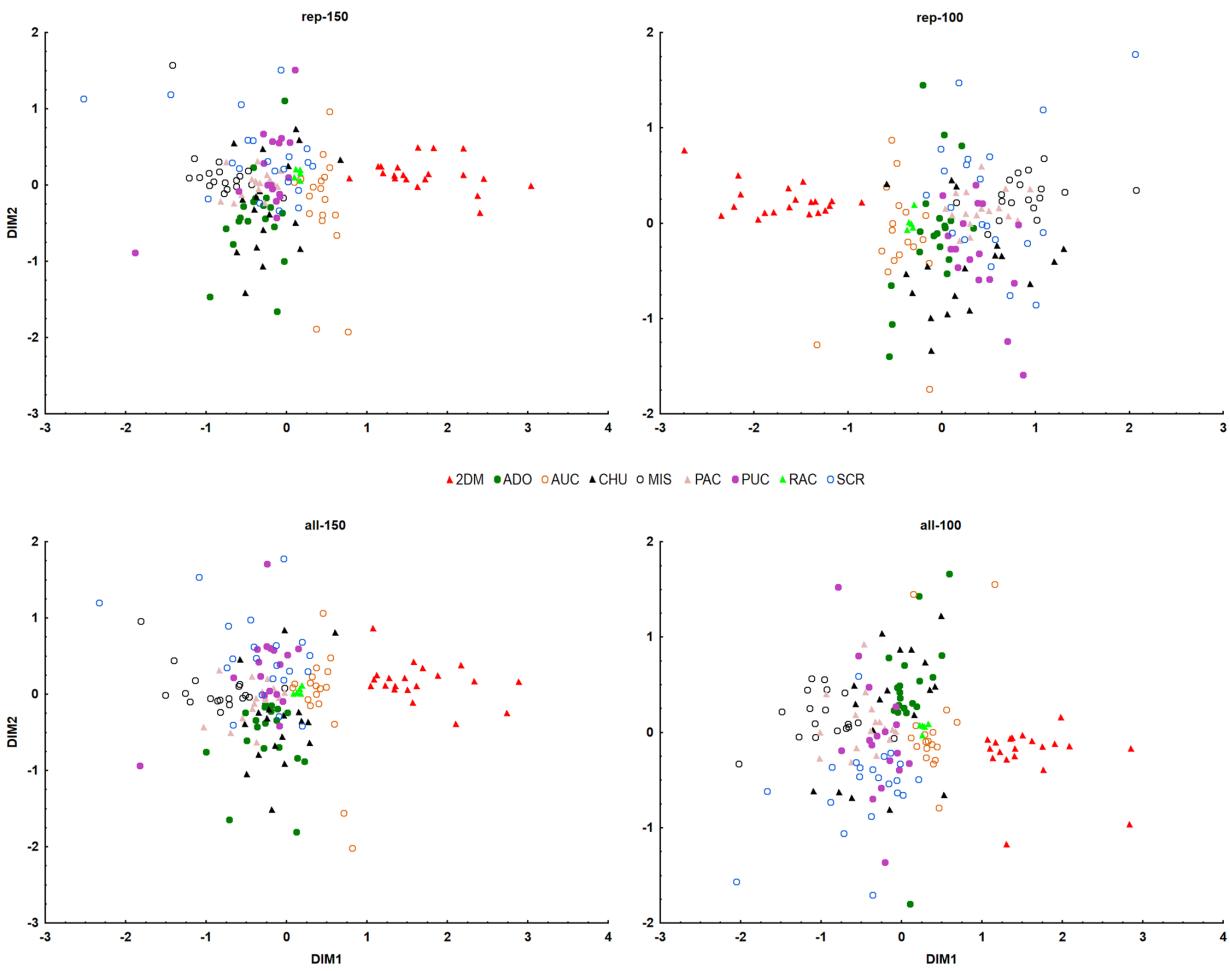
Result of MDS analysis in the form of a 2D projection onto the plane for the “RawGeno” datasets. This graphic projection represents individual samples from Dos de Mayo (2DM) as one cluster and the second cluster consists of all remaining samples from Aguas de Oro (ADO), Aucaloma (AUC), Chumbaquihui (CHU), Mishquiyacu (MIS), Pacchilla (PAC), Pucallpa (PUC), Ramón Castillo (RAC) and Santa Cruz (SCR).

**Fig 5:**
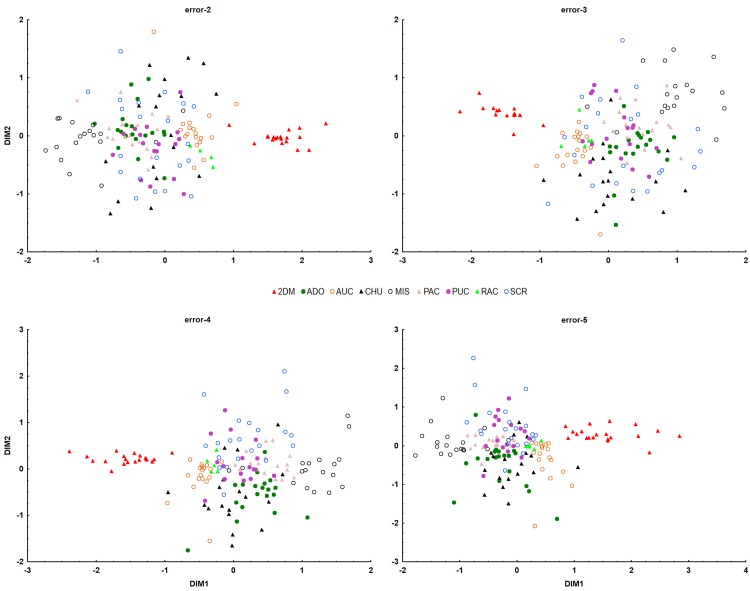
Result of MDS analysis in the form of a 2D projection onto the plane for the “Error” datasets. This graphic projection represents individual samples from Dos de Mayo (2DM) as one cluster and the second cluster consists of all remaining samples from Aguas de Oro (ADO), Aucaloma (AUC), Chumbaquihui (CHU), Mishquiyacu (MIS), Pacchilla (PAC), Pucallpa (PUC), Ramón Castillo (RAC) and Santa Cruz (SCR).

**Fig 6:**
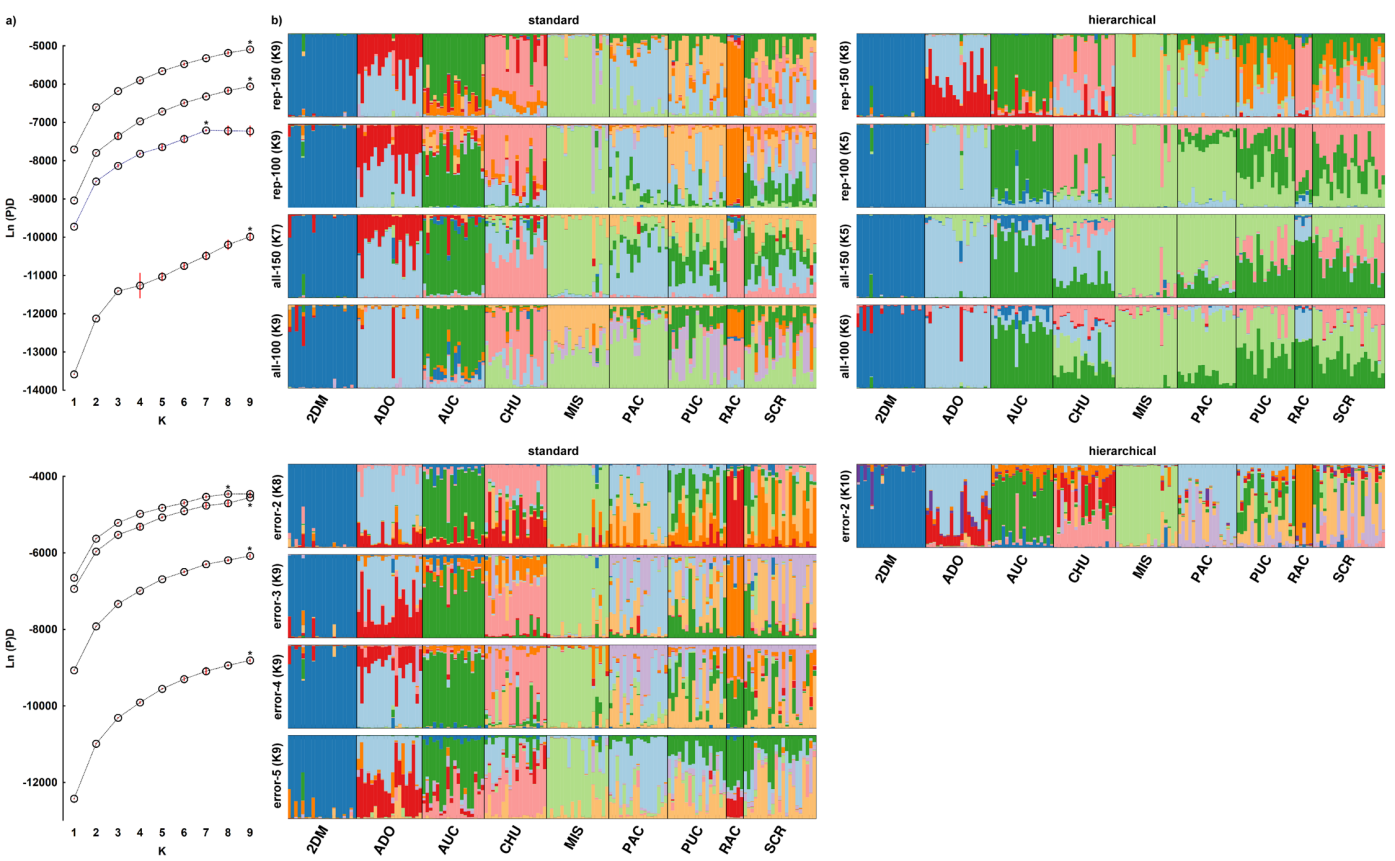
**Mean ± S.D. (red vertical line) Ln(P)D value over 10 replicated runs for each estimated K = 1–9 on the left part of the figure (a) in the case of “standard” analysis.** The chosen K is indicated by * for better clarity. The order of estimation Ln(P)D value for each dataset is equal to the dataset order on the side of graph bars. Please note the different scales of the Ln(P)D axes. The right part of the figure (b) shows a graph of each individual within the appropriate subpopulation indicated by the shortened name. The segmentation of vertical bars by different colors represents the estimated membership of an individual in K inferred clusters. There are two results for each dataset whenever K was estimated differently for the “standard” and “hierarchical” types of STRUCTURE analysis.

Testing three distance matrices with MDS showed that both the Dice and Jaccard coefficients (0.2146–0.2339) seemed to perform better than the simple match coefficient (0.2264–0.2405) according to the stress parameter. A two-dimensional model was selected because, even though the three-dimensional model exhibited better values of the stress parameter, it did not exhibit any improvement in biological interpretation against the two-dimensional model (S6 Table). MDS indicated the existence of only two clearly distinct clusters (Figs 4 and 5). The first cluster consisted of samples from the Dos de Mayo (2DM) locality and the second cluster consisted of all the remaining samples. A similar result was reached using the AMOVA-based K-means clustering method when the assessed parameter was pseudo-F. In cases where the BIC value was used as the criterion for the same method, the preferred number of clusters was nine (S7A and S7B Table). STRUCTURE showed for the “standard” variant K = 9 for all the datasets except datasets error-2 and all-150, where K = 8 and 7, respectively (Fig 6). The situation was more complicated in the case of “hierarchical” analysis because the assessment of the number of clusters varied greatly. Almost all “Error” datasets (error-3, 4, 5) were in agreement with the standard STRUCTURE analysis and showed K = 9, while dataset error-2 showed K = 10, which would mean there were more populations than locations where the samples were collected. In general, the “RawGeno” datasets showed a lower number of populations where K = 5 (rep-100, all-150), 6 (all-100) or 8 (rep-150), respectively. The results of assessment of population structure are summarized in Table 3.

**Table 3:** Summary information about K number estimation by different statistical methods.

Dataset	MDS	AMOVA based K-means clustering		STRUCTURE	
		pseudo-F	BIC	standard	hierarchical
rep-100	2	2	9	9	5
rep-150	2	2	9	9	8
all-100	2	2	9	9	6
all-150	2	2	9	7	5
error-2	2	2	9	8	10
error-3	2	2	9	9	9
error-4	2	2	9	9	9
error-5	2	2	9	9	9

Assigning the individuals to populations according to the sample collection site based on a re-allocating procedure showed that 37 individuals were not assigned to any population and 19 were re-allocated incorrectly in dataset error-2, i.e. 56 individuals (36.6%) in total. In dataset error-3, this number decreased to 26 unassigned individuals and again 19 assigned incorrectly, i.e. 45 individuals (29.41%) in total. The remaining datasets showed that a stable plateau of unassigned individuals ranging among 19–21 and 15–18 samples were misassigned, i.e. 23.42% on average. The lowest rate of correct assignment was typical for the SCR (20–40% individuals according to dataset), PAC (23–47%) and PUC (23–41%) subpopulations in which the highest level of admixture was also detected.

### Genetic diversity

The comparison of differences between the minimal and maximal values across datasets for individual subpopulations showed that the He values always differed by at least ~30% and in some cases even by 45%. A more detailed overview is available in Table 4 showing the values of the basic parameters (He, Φ_PT_, F_ST_, PLP5%). Inbreeding coefficient (F_IS_) varied from 0.0065 to 0.0718 (95% Credible Interval—CI95 from 0.0002 to 0.2059) according to median depending on population and dataset (Table 5).

**Table 4:** Descriptive statistics of each dataset including total number of loci, number of polymorphic loci at the 5% level (#loc_P), proportion of polymorphic loci at the 5% level (PLP) expressed as a percentage, average expected heterozygosity (He), standard error of He (S.E.), fixation index (F_ST_), and F_ST_ analogue for binary markers (Φ_PT_).

Dataset	No. loci	#loc_P	PLP5%	He	S.E.	F_ST_	Φ_PT_
rep-100	154	108	70.1	0.188	0.013	0.235	0.304
rep-150	120	49	40.8	0.179	0.015	0.241	0.301
all-100	275	127	46.2	0.128	0.009	0.203	0.252
all-150	192	100	52.1	0.139	0.012	0.212	0.253
error-2	102	56	54.9	0.153	0.017	0.271	0.323
error-3	108	60	55.6	0.166	0.015	0.266	0.315
error-4	142	82	57.7	0.171	0.014	0.254	0.309
error-5	194	112	57.7	0.173	0.012	0.220	0.258

**Table 5:** Median F_IS_ values of all populations and datasets.

Population	rep-100	rep-150	all-100	all-150	error-2	error-3	error-4	error-5
**2DM**	0.0348	0.0157	0.0379	0.0158	0.0097	0.0089	0.0079	0.0141
**ADO**	0.0119	0.0106	0.0172	0.0093	0.0118	0.0111	0.0086	0.0098
**AUC**	0.0409	0.0420	0.0228	0.0276	0.0455	0.0226	0.0140	0.0561
**CHU**	0.0082	0.0079	0.0089	0.0082	0.0206	0.0151	0.0123	0.0097
**MIS**	0.0350	0.0461	0.0352	0.0451	0.0142	0.0125	0.0234	0.0343
**PAC**	0.0065	0.0067	0.0070	0.0070	0.0113	0.0121	0.0112	0.0621
**PUC**	0.0294	0.0226	0.0218	0.0179	0.0151	0.0142	0.0115	0.0097
**RAC**	0.0163	0.0170	0.0163	0.0166	0.0230	0.0214	0.0188	0.0169
**SCR**	0.0718	0.0629	0.0603	0.0613	0.0163	0.0167	0.0179	0.0498

### Spatial analysis

The assessment of the connection between genetic and geographical distance was performed at the subpopulational level. The Mantel test did not confirm the hypothesis about the occurrence of a spatial genetic pattern (S10 Table) because a statistically significant correlation was not found between genetic and geographical distance for any dataset (r = 0.427–0.501, p = 0.061–0.106). Also, the sPCA method did not show either a global (Gtest: t(max) = 0.1604–0.2107, p = 0.1003–0.3511 according to dataset) or local (Ltest: t(max) = 0.1621–0.2192, p = 0.5528–0.7868 according to dataset) spatial structure (S11 Table). However, analysis by correlogram showed a weak positive autocorrelation for the datasets error-4 (r = 0.118, p = 0.037), rep-150 (r = 0.112, p = 0.044) and all-150 (r = 0.118, p = 0.041) in the distance class 20 km and a weak negative autocorrelation in the distance class 50 km for datasets error-5 (r = -0.095, p = 0.047), rep-100 (r = -0.103, p = 0.034) and all-100 (r = -0.099, p = 0.047). No statistically significant autocorrelations were detected in the other datasets (S12 Table, S3 Fig).

## Discussion

### Error rate analysis

An objective report about the data quality requires a more detailed description of the whole filtering procedure instead of just mentioning numerical value of the genotyping error. Thus, one of our aims was to transparently show each particular step and the procedures of error rate analysis so that anyone could repeat it with an available data file (DOI: 10.5061/dryad.j702j). We can demonstrate this need by comparison our “RawGeno” datasets all-100 and all-150 against the rep-100 and rep-150 dataset. These datasets differ by retained/removed untested bins thus total number of markers (here 121/72). Nonetheless, the software gives the same value of error rate for both datasets, although there is higher uncertainty about data quality in all-100 and all-150 datasets.

Moreover, in a number of published studies, the error rate is just reported without an explanation of how it was calculated [8]. Thus, it is also necessary to be more specific about the genotyping error formula like mismatch error rate [17], Jaccard error rate [17], or Bayesian error rate [53]. For our comparison purposes mismatch error rate formula was chosen because it is the most common approach to date, although it is necessary to take account of some pitfalls connected to this parameter as pointed out by Ley and Hardy [54]. Mismatch error rate tends to keep low quality markers with too high and low frequencies. It also treats equally present peaks and absence peaks matches which means it is prone to error caused by size homoplasy unlike Jaccard error rate.

There is another source of error prevailing in AFLP analysis called homoplasy [55], which could be classified as technical or size homoplasy [21]. Technical homoplasy is caused by the incorrect definition of bins. This may lead to opposite extremes, i.e. either oversplitting (very narrow bins) or artificial similarity of non-homologous fragments (overly wide bins). Therefore, bin width is an important criterion during the processing of fluorescent signals [31, 28]. It was one of the reasons why RawGeno was chosen, because it offers better binning flexibility than GeneMapper by simply setting several parameters. Herrmann et al. [28] stated that the most important parameter is MAXBIN, which determines the maximum width of bin, because it has the greatest influence on F_ST_. The recommended values of this parameter were over 1 bp and ideally 2 bp or more. We chose a compromise value of 1.5 bp, leading to the elimination of bin oversplitting and a reduction in technical homoplasy in the ideal case.

The second type of homoplasy, size homoplasy [56] is caused by similar/same migration rate of fragments from different loci, thus probably loci with different sequences and evolutionary history. This is why AFLP is mostly used for interrogation of intraspecies genetic variability. The risk of size homoplasy increases with taxonomic distance. We minimized the error caused by size homoplasy, only counting fragments bigger than 100 bp because more than one third of the fragments below this threshold value can be non-homologous [56]. Further, Vekemans et al. [27] found a negative correlation between the size of fragments and their frequency in the population as an indicator of the presence of size homoplasy. A statistically significant but weak correlation (r = - 0.13, p = 0.028) was found in only one dataset (all-100), which contained the highest absolute number of markers and the highest number of markers without error rate testing. From the comparison of all eight datasets, it was obvious that the “Error” datasets were better than the “RawGeno” datasets, which oscillated around the edge of a statistically significant correlation (S4A and S4B Table). Simultaneously, testing of other size thresholds (150, 200, 250, and above 300 bp) was performed (S5 Table). The presence of size homoplasy was even detected in three cases for fragments over 250 and 300 bp (p < 0.05). Apparently, this is a phenomenon that cannot be easily eliminated according to size criteria.

The multiple datasets approach covering various levels of the error rate, the influence of software choice, and the parameter settings were included as well as other aspects connected with the error rate, such as homoplasy. The purpose was not a comparison of programs or their algorithms but rather to show the limitations of our study. It is difficult to choose objectively the best dataset because we did not detect some kind of "break point" as in the case of Zhang and Hare study [10], but there a pattern was seen across the datasets.

Firstly, the "Error" datasets gave more consistent results for various kinds of tests than "RawGeno" datasets and, secondly, a lower number of markers as a result of too stringent selection criteria limit the power of some statistical approaches to obtain the best estimation of true population structure. For a conservative approach with mismatch error rate, we suggest using RawGeno only for binning purpose, thus without a reproducibility test, then (optionally) do some kind of marker preliminary screening using not too stringent criteria for error rate estimator, and finish the analysis for marker choice in some software such as AFLPScore [20] or scanAFLP [57] dealing with rfu profiles. It is also possible to use newer alternative approach published by Ley and Hardy [54] enabling the elimination of weaknesses of mismatch error rate where a marker is treated as a phenotypic trait influenced by both genetic and non-genetic factors. However, this approach requires a larger number of replicates and initial number of bins.

### Comparison of clustering methods

In order to understand the population structure of *P*. *volubilis*, several statistical methods were employed. The first technique (MDS) possesses some advantages in comparison with more popular Bayesian clustering methods [58]. For the final MDS, a matrix based on the Jaccard coefficient was selected because it is less influenced by potential homoplasy [30]. However, from a statistical point of view, the quality of all two-dimensional models was very low, regardless of the coefficient used. In all cases, stress values were above 0.2, indicating poor fitting of the data by the relevant model [33]. Three-dimensional models led to a decrease in stress value, but they did not offer any improvement in biological interpretation. As is shown, MDS was able to capture only the highest hierarchical structure, namely by distinguishing samples from the locality of Dos de Mayo (2DM) from the others (Figs 4 and 5).

The other method combining AMOVA with the K-means hierarchical clustering method revealed very different estimations for the number of clusters, depending on the summary statistics used (pseudo-F or BIC). The result of pseudo-F was the same as in MDS, i.e. two clusters, but the BIC criterion showed nine clusters (S7A and S7B Table). Cluster estimations were the same for all datasets and thus, surprisingly, neither the software settings nor the error rate had a significant influence on the result. Meirmans [34] mentioned that the predictive value of pseudo-F and BIC parameters is strongly influenced by the reproductive mode of the organism. The pseudo-F parameter is supposed to be a better estimator in cases of random mating and BIC should be preferred in cases of non-random mating or in the presence of significant population structure [34]. As discussed in the “Population structure” section, in the case of *P*. *volubilis*, the final effect of selection for seed size is similar to non-random mating and the preferred parameter should be BIC.

The last comparative method was a model approach based on the Bayesian clustering algorithm. The “standard” type of analysis, corresponding to the original methodology proposed by authors of program STRUCTURE [37], showed fairly robust results across datasets; only in two cases was a value found other than K = 9 found (Table 3). It is obvious that the error rate or various parameter settings did not play an important role. In the case of the dataset error-2 (K = 8), an impact of the lower number of markers retained for analysis could be assumed. One can argue that the difference in the number of markers between the error-2 and error-3 datasets is not significant (102 vs. 108), but the result is also dependent on which specific loci were retained/discarded and their informational value in terms of the structural signal. Moreover, there was a minimal difference in the average LnP(D) estimation for K = 8 and 9 (-4460.04 vs. -4460.33) in the error-2 dataset, but there was higher variation for K = 9 (SD = 26.06 vs. 43.61) and therefore eight clusters were selected. The dataset all-150 showed K = 7, while the dataset all-100 gave the best estimation for K = 9. The only difference was in the total number of markers (275 vs. 192) and thus we suppose that the structural signal was higher than the background noise in the all-100 dataset but not in the all-150 dataset.

The “hierarchical” variant of analysis in STRUCTURE proposed by Coulon et al. [42], showed the highest variability in the estimation of K according to the dataset. The “Error” datasets were consistent and for all the “Error” datasets an estimation of K = 9, was achieved except for error-2 with K = 10 (Table 3). A higher number of clusters than the number of collection sites were considered too unlikely due to the method of *P*. *volubilis* cultivation and the size of the plots. Therefore, the imbalance in the K estimation can be attributed to an unsuitable number of markers.

A different situation occurred in the “RawGeno” datasets where the estimation of K ranged from K = 5 (rep-100, all-150) to K = 6 (all-100) and K = 8 (rep-150). There were perhaps the most apparent differences between “RawGeno” datasets. Hierarchical analysis at each step typically showed an estimation according to ΔK = 2 or 3 (S2 Fig), when the gradually decreasing number of individuals, and thus also markers, may be more affected by noise in the data, which exceeded the structural signal in less differentiated groups such as PUC, PAC and SCR.

### Population structure and genetic diversity

A comparison of clustering methods indicates with high probability the existence of nine genetically differentiated clusters, which largely coincide with the locations of sample collection. Only MDS or parameter pseudo-F in the AMOVA-based K means a clustering method preferred two clusters. It raised the question what was the cause of high genetic differentiation into subpopulations occurring in the relatively small area about 70 km^2^. On the basis of the available information we suppose strong anthropogenic influence determining the magnitude of several evolutionary forces like selection, genetic drift and gene flow on population structure of *P*. *volubilis*.

To fully realize how selection and other forces diversified one subpopulation from another, it is necessary take into consideration how and how long *P*. *volubilis* has been cultivated. Farmers cultivating this crop do not purposely carry out cross-breeding and open pollination occurs. However, from the obtained seeds, the biggest ones are selected as a basis for the next generation of plants. From the breeding perspective, this is not such an efficient method (so-called selection after flowering) and it is slower than other methods (e.g. pedigree or recurrent selection). Nevertheless, selection for a certain phenotype is happening as well as on genes or control regions influencing this phenotype. Simultaneously, genetic hitchhiking of associated regions is occurring and leads to the fixation of alleles positively influencing required trait(s) and alleles of neutral loci close to these genes which supports structuring into local subpopulations, as indicated by the Φ_PT_ or F_ST_ values (Table 4). This is a typical consequence of domestication and has been noted in many crops [59].

The selection process probably started more than 800–900 years ago because cultivation of this crop is known from time of pre-Inca tribes and Inca Empire [1, 60]. Despite the relatively long generation cycle of about 6–8 years, there was a sufficient number of generations for substantial differentiation between the localities thanks to genetic drift accelerated by selection for certain phenotype. Moreover, sacha inchi has limited ability for seed or pollen dispersion. The pollen is only dispersed over a short distance (approx. 100 m) and seeds can be occasionally distributed by rodents (pers. obs.). Further, it is necessary to take into account a specific requirement for growth conditions, with the most important ones being sunny places [61]. In the forested areas of the Peruvian Amazon, these places are represented by village fields and in the present time the wild form of sacha inchi practically does not occur. Thus, there is a lot of evidence of the limited dispersion capability of *P*. *volubilis*, even for short distance in the range of several kilometers, and thus support for the hypothesis of low gene flow.

The idea of low gene flow is in contrast with the graphical result of STRUCTURE analysis which revealed unexpectedly high levels of admixture between several subpopulations (PUC, PAC and SCR; see Fig 6). Also, the assignment test confirmed that 11–12% of individuals were assigned to a different subpopulation than according to locality and a similar percentage of individuals were not assigned to any of the studied subpopulations. Apparently, there is some mechanism which allows gene flow between the localities. Natural gene flow is obviously limited, although anthropogenic transfer of this economically important plant might play a role here. Besides the effect caused by migration of people from one village to another, taking sacha inchi seeds with them, another possibility is exchange of seeds on the market. Seeds that are sold are mixtures originating from different areas (pers. com.), and it is therefore unknown as to which plants will grow in the field according to the place of origin.

Anthropogenic influence, causing differentiation of subpopulations in one way and facilitating gene flow between the localities despite the natural barriers in another way, could explain why the MDS (or AMOVA based K-means clustering) method was able to clearly distinguish only plants from Dos de Mayo. This locality is poorly accessible, the most distant from the other localities, not currently inhabited, and plants growing there are therefore isolated. We do not know how much time has passed since the last villager leaved this place, but it was undoubtedly long enough for elimination of gene flow through anthropogenic transfer of seeds mixture. This example could be used as another indirect evidence of strong influence on population structure by human being. Another insight into observed population structure and connectivity between the sites could provide analysis both spatial and genetic data.

The primary idea is usually based on the hypothesis that individuals as well as populations that are geographically more distant will be even less genetically similar, and vice-versa [62–64]. In the case of *P*. *volubilis*, rather than a demonstration of isolation by distance (IBD), differentiation can be expected through reduced gene flow between subpopulations due to barriers such as the rainforest or mountains. However, no spatially oriented test showed a statistically significant result (see S10–S12 Tables) with the exception of a correlogram of several datasets (all-150, rep-150 and error-4). This leads to an interesting situation when AMOVA revealed an unusually high proportion of variability between subpopulations (Φ_PT_ = 25–32% according to dataset (see Table 4), but with no statistically significant spatial structure.

We suppose this is another clue for prevailing anthropogenic factors among others. When we accept this idea then the values of the pairwise Φ_PT_ parameter can also serve as an indicator of the socio-economical connectivity between the localities. It was found that the subpopulations 2DM, ADO, AUC, MIS and RAC showed a high level of differentiation toward any of subpopulations; conversely, between PUC, PAC and SCR the Φ_PT_ values were low, although statistically significant (S8A–S8H Table). Unfortunately, we cannot provide a satisfactory explanation for the higher connectivity of these localities against the others because we lack information about the underlying history of this area or human migration. As far as we know, the plants from Pucallpa (PUC) and Pacchilla (PAC) are cultivated for commercial purposes and maybe the traditional "seed size selection" model followed by local villagers is not applicable here. We are expecting the faster generation cycle and demands for a higher amount of seeds. At present, it is not possible to buy any bred cultivars and the only source are mixtures of seeds from the markets. The reason behind the high admixture level in Santa Cruz (SCR) remains unclear.

As we mentioned earlier, despite long-term phenotypic selection, *P*. *volubilis* is not considered to be a fully domesticated plant. Our former plan was analysis of “pure” wild *Plukenetia* plants, but we have found that its occurrence is so intertwined with village fields that it is not possible to set a clear border between the plants cultivated in village fields and “naturally” occurring plants. According to our observation, the plants in the studied area have great potential as a promising genotype source for breeding because there is a high differentiation level among the subpopulation and sufficient variability within (almost) each subpopulation according to He etc. (Table 4). The lowest polymorphism and variability was only found in the RAC subpopulation, followed by 2DM in many datasets. In the case of RAC, this is a consequence of sampling because in this locality it was impossible to find a large number of plants and this skewed the results. The lower diversity 2DM subpopulation is probably due to the isolation of the locality and thus low or zero gene flow.

A further insight into the subpopulation structure is provided by an estimation of F_IS_, where average values of F_IS_ across the datasets were rather similar (1–2%), but between the subpopulations varied more widely (0.5–7%). When we omit the technical issues connected with AFLP like parameter estimation based on dominant type of markers, genotyping error, homoplasy, coverage of different genome part, and frequent violation of assumption about linkage equilibrium [49], then possible biological explanations of inbreeding include partial self-pollination, biparental inbreeding, and selection, or a combination of these factors. Sacha inchi is considered to be an allogamous plant with a dichogamic mechanism against selfing. The efficiency of this mechanism could be reduced by environmental conditions such a temperature as is known in other plants like avocado [65], and thus we can expect some degree of selfing (pers. com.), but unfortunately there are no data available. Also, biparental inbreeding could occur in some subpopulations due to small local population size and limited range of pollen dispersion [66]. Moreover, as we already know, phenotypic selection is happening. We can conclude that the observed level of inbreeding is probably caused by several factors and, despite this, it is relatively low even for small subpopulations like RAC.

## Conclusion

The comparison of eight datasets showed that it is better to perform variant multiple data filtering by combining several different approaches. Despite the understandable differences, the “Error” datasets (error-2, error-3, error-4, and error-5), showed more consistent results than “RawGeno” datasets (rep-100, rep-150, all-100, and all-150). The estimation of clusters was independent of reproducibility of the underlying dataset. The exception was hierarchical analysis in STRUCTURE where, thanks to the gradual reduction in the number of individuals and markers, the background noise was predominant over the structural signal in datasets with higher error rates. However, noticeable differences were found in the genetic diversity estimation between datasets. Data quality considerably influenced estimates of genetic diversity, which is a concern for conservation genetics.

*P*. *volubilis* represents a traditional Peruvian oil crop for human nutrition. Recently, a growing interest in this crop has been noted within the food and pharmaceutical industry. Breeding of sacha inchi allows for improvements in the required characteristics, but for the successful creation of varieties it is necessary to learn about its genetic diversity and variability. Our study showed that, due to long-term phenotypic selection and low gene flow, *P*. *volubilis* exists as very isolated subpopulations corresponding with the sampling localities. The degree of differentiation between the subpopulations is probably due more to socio-economic connectivity among the sites than to biological connection, because seeds are transported over different distances. Using the AFLP technique and appropriate filtering data and estimation of error rate can be successfully applied for genetic structure estimation. For the first stage of the breeding process, it would be interesting to create a collection of samples originating from neighboring localities. However, questions remain regarding the degree of variability within loci affected by selection for the desired phenotype. Answers could be provided by association studies aiming to identify these loci.

## Supporting information

S1 Text AFLP protocol. (DOCX)

S1 Table Information about collection sites and the number of samples. (DOCX)

S2 Table Number of bins for each tested primer set with respect to selective the PCR variant (T58 or T64) and rfu threshold (100 or 150 rfu). (DOCX)

S3 Table Information about scoring parameters in AFLPScore v1.4b for the “Error” datasets from error-2 dataset to the error-5 datasets. (DOCX)

S4 Table Detection of size homoplasy for the “RawGeno” and “Error” datasets based on a linear regression model describing the correlation between the size of the fragments and their frequency in the population. The correlation coefficient and p-value for each primer set and concatenated matrix are shown. Statistically significant results are highlighted.
(DOCX)

S5 Table Size homoplasy (SH) decomposition. Detection of SH based on a linear regression model with different criteria for the minimum fragment size (150, 200, 250 and 300 bp). The correlation coefficient and p-value for each dataset are shown. Statistically significant results are highlighted.
(DOCX)

S6 Table Comparison of several similarity coefficients for two- and three-dimensional MDS models based on the stress parameter. (DOCX)

S7 Table Optimal number of K clusters according to the pseudo-F and Bayesian Information Criterion (BIC) for the “RawGeno” and “Error” datasets. The best clustering according to pseudo-F (highest value) and BIC (lowest value) is highlighted.
(DOCX)

S8 Table Pairwise population Φ_PT_ values. Φ_PT_ values are shown below diagonal and p-values based on 9999 permutation are shown above diagonal. (DOCX)

S9 Table Genetic diversity (He) of each subpopulation. (DOCX)

S10 Table Spatial analysis to test the statistical relationship between genetic and geographical distance by the Mantel test with 9999 random permutations. The correlation coefficient and p-value are shown.
(DOCX)

S11 Table Spatial analysis in the frame of sPCA where the presence of a global and/or local spatial pattern was tested by the G and L test. (DOCX)

S12 Table Numerical results of spatial autocorrelation analysis using the random permutation procedure for seven equidistant classes (10 km). The genetic distance matrix was based on Φ_PT_ values. Statistically significant results are highlighted.
(DOCX)

S1 Fig Run order according to Ln(P)D for the chosen K value. The gray box highlights removed low quality runs. The number of retained runs is written in brackets together with the K value and the type of STRUCTURE analysis (“standard” and/or “hierarchical”). Please note the different scales of the Ln(P)D axes.
(TIF)

S2 Fig Result of the “hierarchical” type of STRUCTURE analysis for the “RawGeno” and “Error” datasets. Estimation of the K number according to ΔK for each round of hierarchical analysis and the number of individuals within the cluster is shown. Red colored clusters represent clusters which could not be divided any further.
(TIF)

S3 Fig Graphical results of the spatial autocorrelation analysis in the form of correlograms for the “RawGeno” and “Error” datasets. The calculated r value (blue line) is shown with upper (U) and lower (L) bounds of the 95% confidence interval (red lines).
(TIF)
