# Supplementary material for: Dealing with AFLP genotyping errors to reveal genetic structure in Plukenetia volubilis (Euphorbiaceae) in the Peruvian Amazon
Source: PLoS One. 2017 Sep 14;12(9):e0184259. doi: 10.1371/journal.pone.0184259 (PMC5598967; doi:10.1371/journal.pone.0184259)
Supplement: S1 Text — (DOCX) [file pone.0184259.s001.docx]

**S1 Text. AFLP protocol**

Genomic DNA restriction and adapter ligation was performed in a single step procedure. The reaction mixture with a total volume 12.5 μl contained 100 ng DNA, 1x T4 DNA ligase buffer (Thermo Fisher Scientific), 1U T4 DNA ligase (Thermo Fisher Scientific), 5U *Eco*RI (Thermo Fisher Scientific), 1 U *Mse*I (New England Biolabs), 55 ng BSA, 50 μM NaCl_2_, 0.05 μM *Eco*RI adapter and 0.5 μM *Mse*I adapter. The reaction mixture was incubated for 3 hours at 37°C and then diluted 1:1 in 0.1x TE buffer.

Preselective amplification was performed in a reaction mixture with a total volume of 25 μl. The concentrations of the other components were: 3 μl cleaved + ligated DNA, 1U *Taq* polymerase (Roche), 1x KCl buffer with 1.5 mM MgCl_2_, 0.12 mM dNTP, 0.2 μM for both *Eco*RI+1 (5’GACTGCGTACCAATTC**A**3’) and *Mse*I+1 primer (5’GATGAGTCCTGAGTAA**C**3’). The temperature profile included 1x pre-denaturation at 94°C for 120 s, followed by 20 cycles of denaturation at 94°C for 30 s, annealing at 56°C for 60 s and elongation at 72°C for 60 s. A ramp rate of 1°C/s was set for all steps of PCR. The obtained amplicons were consequently diluted 1:9 in 0.1x TE buffer.

Selective amplification was performed in a reaction mixture with a total volume of 12.5 μl. The reaction mixture contained 2.5 μl products of preselective amplification and 1U of *Taq* polymerase (Roche). The concentrations of the other components were: 1x KCl buffer with 1.5 mM MgCl_2_, 0.2 mM dNTP and 0.4 μM for both types of selective primers *Eco*RI+3/*Mse*I+3. All tested *Eco*RI primers were fluorescently labeled with one of four fluorophores – 6FAM, VIC, NED or PET. As a part of the optimization procedure and comparative analysis, two temperature profiles were tested, which differed mainly in annealing temperature (T_a_) or in the application touch-down PCR (TD-PCR). The first variant (T58) with TD-PCR included 1x pre-denaturation at 94°C for 120 s followed by 10 cycles of denaturation at 94°C for 30 s, annealing at 65°C with 1°C/cycle for 30 s and elongation at 72°C for 60 s. Another 30 cycles remained the same, but T_a_ was set at 58°C for 30 s. The second variant (T64), which was chosen for the analysis itself and was tested for all 64 possible combinations given by the number of primers was comprised of 1x pre-denaturation at 94°C for 120 s followed by 25 cycles of denaturation at 94°C for 20 s, annealing at 64°C for 60 s and elongation at 72°C for 120 seconds. To eliminate so called split-peaks, an incubation was performed at 72°C for 1,800 s. All amplifications were carried out in a C1000 thermocycler (Bio-Rad) and the result of each individual step of the AFLP protocol (ligation + digestion, preselective and selective PCR) was checked by electrophoretic separation on a 1% agarose gel. As a part of the optimization, the amount of DNA for selective amplification (products were diluted 1:4 or 1:9 in low TE buffer) and the amount of polymerase (0.5 or 1 U) were also tested.
