# Supplementary material for: Dealing with AFLP genotyping errors to reveal genetic structure in Plukenetia volubilis (Euphorbiaceae) in the Peruvian Amazon
Source: PLoS One. 2017 Sep 14;12(9):e0184259. doi: 10.1371/journal.pone.0184259 (PMC5598967; doi:10.1371/journal.pone.0184259)
Supplement: S1 Table — (DOCX) [file pone.0184259.s002.docx]

**S1 Table.** Information about collection sites and the number of samples.

| **Locality** | **Abbrev.** | | **Latitude** | **Longitude** |  | **Altitude [m]** | **N** |
| --- | --- | --- | --- | --- | --- | --- | --- |
| 1. Dos de Mayo | 2DM |  | 6°47.573' S | 76°32.108' W |  | 335 | 22 |
| 2. Aguas de Oro | ADO |  | 6°17.570' S | 76°39.200' W |  | 385 | 20 |
| 3. Aucaloma | AUC |  | 6°24.816' S | 76°26.143' W |  | 740 | 18 |
| 4. Chumbaquihui | CHU |  | 6°21.991' S | 76°34.504' W |  | 364 | 20 |
| 5. Mishquiyacu | MIS |  | 6°21.673' S | 76°34.998' W |  | 470 | 20 |
| 6. Pacchilla | PAC |  | 6°25.694' S | 76°27.729' W |  | 703 | 20 |
| 7. Pucallpa | PUC |  | 6°25.676' S | 76°34.689' W |  | 455 | 21 |
| 8. Ramón Castillo | RAC |  | 6°35.244' S | 76°07.884' W |  | 210 | 5 |
| 9. Santa Cruz | SCR |  | 6°36.803' S | 76°44.452' W |  | 425 | 23 |
