# Supplementary material for: Dealing with AFLP genotyping errors to reveal genetic structure in Plukenetia volubilis (Euphorbiaceae) in the Peruvian Amazon
Source: PLoS One. 2017 Sep 14;12(9):e0184259. doi: 10.1371/journal.pone.0184259 (PMC5598967; doi:10.1371/journal.pone.0184259)
Supplement: S2 Table — (DOCX) [file pone.0184259.s003.docx]

**S2a Table.** Number of bins for each tested primer set with respect to selective the PCR variant (T58 or T64) and rfu threshold (100 or 150 rfu).

| **Variant** | **Dye** | **Primer** | | **M17** | **M18** | **M19** | **M20** | **M21** | **M22** | **M23** | **M24** |
| --- | --- | --- | --- | --- | --- | --- | --- | --- | --- | --- | --- |
|  |  |  |  | **CAA** | **CAC** | **CAG** | **CAT** | **CTA** | **CTC** | **CTG** | **CTT** |
| T58 | 6FAM | **E01** | **ACT** | 41 | 64 | 50 | 20 | 33 | 49 | 12 | 25 |
|  | VIC | **E02** | **ACA** | 43 | 30 | 26 | 10 | 40 | 34 | 21 | 49 |
| 100 rfu | NED | **E03** | **AAC** | 37 | 25 | 30 | 27 | 27 | 19 | 17 | 48 |
|  | PET | **E04** | **ACC** | 28 | 54 | 44 | 44 | 4 | 11 | 20 | 5 |
| T58 | 6FAM | **E01** | **ACT** | 23 | 61 | 41 | 12 | 22 | 33 | 4 | 19 |
|  | VIC | **E02** | **ACA** | 34 | 15 | 13 | 44 | 31 | 22 | 10 | 34 |
| 150 rfu | NED | **E03** | **AAC** | 25 | 18 | 16 | 19 | 16 | 15 | 7 | 42 |
|  | PET | **E04** | **ACC** | 17 | 45 | 36 | 36 | 3 | 10 | 15 | 2 |

**S2b Table.** Number of bins for each tested primer set with respect to the selective PCR variant (T58 or T64) and rfu threshold (100 or 150 rfu).

| **Variant** | **Dye** | **Primer** | | **M17** | **M18** | **M19** | **M20** | **M21** | **M22** | **M23** | **M24** |
| --- | --- | --- | --- | --- | --- | --- | --- | --- | --- | --- | --- |
|  |  |  |  | **CAA** | **CAC** | **CAG** | **CAT** | **CTA** | **CTC** | **CTG** | **CTT** |
| T64 | 6FAM | **E01** | **ACT** | 63 | 66 | 57 | 67 | 55 | 72 | 69 | 49 |
|  | VIC | **E02** | **ACA** | 28 | 55 | 61 | 56 | 75 | 40 | 65 | 54 |
|  | NED | **E03** | **AAC** | 50 | 45 | 58 | 55 | 27 | 29 | 34 | 44 |
|  | PET | **E04** | **ACC** | 45 | 28 | 36 | 80 | 25 | 36 | 34 | 6 |
| 100 rfu | 6FAM | **E05** | **AGC** | 22 | 12 | 28 | 33 | 20 | 18 | 18 | 3 |
|  | VIC | **E06** | **AAG** | 19 | 15 | 14 | 14 | 20 | 13 | 16 | 16 |
|  | NED | **E07** | **AGG** | 16 | 21 | 9 | 16 | 12 | 37 | 13 | 21 |
|  | PET | **E08** | **ACG** | 29 | 51 | 40 | 51 | 48 | 25 | 24 | 21 |

**S2c Table.** Number of bins for each tested primer set with respect to the selective PCR variant (T58 or T64) and rfu threshold (100 or 150 rfu).

| **Variant** | **Dye** | **Primer** | | **M17** | **M18** | **M19** | **M20** | **M21** | **M22** | **M23** | **M24** |
| --- | --- | --- | --- | --- | --- | --- | --- | --- | --- | --- | --- |
|  |  |  |  | **CAA** | **CAC** | **CAG** | **CAT** | **CTA** | **CTC** | **CTG** | **CTT** |
| T64 | 6FAM | **E01** | **ACT** | 57 | 65 | 47 | 66 | 52 | 68 | 66 | 47 |
|  | VIC | **E02** | **ACA** | 14 | 51 | 54 | 44 | 67 | 37 | 61 | 39 |
|  | NED | **E03** | **AAC** | 46 | 40 | 56 | 52 | 19 | 26 | 27 | 41 |
|  | PET | **E04** | **ACC** | 39 | 22 | 28 | 76 | 11 | 32 | 19 | 3 |
| 150 rfu | 6FAM | **E05** | **AGC** | 15 | 7 | 14 | 20 | 11 | 11 | 10 | - |
|  | VIC | **E06** | **AAG** | 12 | 13 | 8 | 11 | 13 | 7 | 12 | 7 |
|  | NED | **E07** | **AGG** | 10 | 10 | 6 | 6 | 6 | 27 | 8 | 14 |
|  | PET | **E08** | **ACG** | 17 | 41 | 30 | 43 | 44 | 13 | 20 | 8 |
