# Supplementary material for: Dealing with AFLP genotyping errors to reveal genetic structure in Plukenetia volubilis (Euphorbiaceae) in the Peruvian Amazon
Source: PLoS One. 2017 Sep 14;12(9):e0184259. doi: 10.1371/journal.pone.0184259 (PMC5598967; doi:10.1371/journal.pone.0184259)
Supplement: S3 Table — (DOCX) [file pone.0184259.s004.docx]

**S3a Table.** Information about scoring parameters in AFLPScore v1.4b for the error-2 dataset.

| **Primer set** | | **Locus selection threshold [rfu]** | **Phenotype calling threshold [rfu] or [%]** | **Bayesian error rate ε_1.0_ [%]** | **Bayesian error rate ε_0.1_ [%]** | **Mismatch error rate [%]** | **Number of loci** |
| --- | --- | --- | --- | --- | --- | --- | --- |
|  |  |  |  |  |  |  |  |
|  |  |  |  |  |  |  |  |
| E02 ACA | M23 CTG | 400 | 150 rfu | 7.01 | 0.72 | 3.75 | 25 |
| E07 AGG | M19 CAG | 50 | 400 rfu | 13.12 | 0.20 | 2.21 | 17 |
| E04 ACG | M21 CAT | 500 | 250 rfu | 12.19 | 0.20 | 2.60 | 12 |
| E01 ACT | M21 CTA | 400 | 50 rfu | 0.98 | 0.61 | 0.00 | 6 |
| E02 ACA | M21 CTA | 300 | 100 rfu | 1.96 | 1.22 | 2.08 | 6 |
| E03 AAC | M20 CAT | 400 | 150 rfu | 2.69 | 2.02 | 2.08 | 3 |
| E04 ACC | M17 CAA | 250 | 25% | 1.30 | 0.26 | 0.00 | 8 |
| E05 AGC | M19 CAG | 150 | 10% | 11.12 | 0.81 | 4.58 | 30 |
| E07 AGG | M17 CAA | 400 | 20% | 3.19 | 0.97 | 2.50 | 5 |
| E08 ACG | M22 CTC | 200 | 50 rfu | 6.72 | 0.61 | 3.13 | 12 |
|  |  |  | **Mean** | 6.03 | 0.76 | 2.29 | 12 |
|  |  |  | **Median** | 4.95 | 0.67 | 2.35 | 10 |
|  |  |  | **Total** | - | - | - | 124 |

**S3b** **Table.** Information about scoring parameters in AFLPScore v1.4b for the error-3 dataset.

| **Primer set** | | **Locus selection threshold [rfu]** | **Phenotype calling threshold [rfu] or [%]** | **Bayesian error rate ε_1.0_ [%]** | **Bayesian error rate ε_0.1_ [%]** | **Mismatch error rate [%]** | **Number of loci** |
| --- | --- | --- | --- | --- | --- | --- | --- |
|  |  |  |  |  |  |  |  |
|  |  |  |  |  |  |  |  |
| E02 ACA | M23 CTG | 400 | 150 rfu | 7.01 | 0.72 | 3.75 | 25 |
| E07 AGG | M19 CAG | 200 | 300 rfu | 15.69 | 0.24 | 2.63 | 19 |
| E04 ACG | M21 CAT | 250 | 20% | 5.91 | 0.53 | 2.78 | 9 |
| E01 ACT | M21 CTA | 400 | 30% | 4.83 | 0.89 | 3.13 | 6 |
| E02 ACA | M21 CTA | 300 | 10% | 2.89 | 1.24 | 2.50 | 5 |
| E03 AAC | M20 CAT | 400 | 150 rfu | 2.69 | 2.02 | 2.08 | 3 |
| E04 ACC | M17 CAA | 100 | 20% | 7.58 | 0.56 | 3.26 | 23 |
| E05 AGC | M19 CAG | 300 | 10% | 7.01 | 1.03 | 4.17 | 21 |
| E07 AGG | M17 CAA | 500 | 15% | 4.73 | 0.95 | 3.13 | 4 |
| E08 ACG | M22 CTC | 200 | 50 rfu | 6.72 | 0.61 | 3.13 | 12 |
|  |  |  | **Mean** | 6.51 | 0.88 | 3.05 | 13 |
|  |  |  | **Median** | 6.32 | 0.81 | 3.13 | 11 |
|  |  |  | **Sum** | - | - | - | 127 |

**S3c Table.** Information about scoring parameters in AFLPScore v1.4b for the error-4 dataset.

| **Primer set** | | **Locus selection threshold [rfu]** | **Phenotype calling threshold [rfu] or [%]** | **Bayesian error rate ε_1.0_ [%]** | **Bayesian error rate ε_0.1_ [%]** | **Mismatch error rate [%]** | **Number of loci** |
| --- | --- | --- | --- | --- | --- | --- | --- |
|  |  |  |  |  |  |  |  |
|  |  |  |  |  |  |  |  |
| E02 ACA | M23 CTG | 400 | 200 rfu | 9.14 | 0.68 | 4.09 | 29 |
| E07 AGG | M19 CAG | 300 | 250 rfu | 19.83 | 0.24 | 3.82 | 18 |
| E04 ACG | M21 CAT | 200 | 50 rfu | 6.68 | 1.04 | 4.41 | 17 |
| E01 ACT | M21 CTA | 300 | 100 rfu | 8.51 | 0.31 | 4.17 | 9 |
| E02 ACA | M21 CTA | 150 | 50 rfu | 7.55 | 0.53 | 3.75 | 10 |
| E03 AAC | M20 CAT | 250 | 20% | 3.95 | 2.99 | 4.17 | 6 |
| E04 ACC | M17 CAA | 50 | 50 rfu | 14.00 | 0.36 | 3.98 | 33 |
| E05 AGC | M19 CAG | 400 | 10% | 7.00 | 1.00 | 3.85 | 13 |
| E07 AGG | M17 CAA | 200 | 15% | 4.96 | 1.34 | 4.17 | 12 |
| E08 ACG | M22 CTC | 150 | 20% | 9.15 | 0.54 | 3.95 | 19 |
|  |  |  | **Mean** | 9.08 | 0.90 | 4.03 | 17 |
|  |  |  | **Median** | 8.03 | 0.61 | 4.04 | 15 |
|  |  |  | **Sum** | - | - | - | 166 |

**S3d Table.** Information about scoring parameters in AFLPScore v1.4b for the error-5 dataset.

| **Primer set** | | **Locus selection threshold [rfu]** | **Phenotype calling threshold [rfu] or [%]** | **Bayesian error rate ε_1.0_ [%]** | **Bayesian error rate ε_0.1_ [%]** | **Mismatch error [%]** | **Number of loci** |
| --- | --- | --- | --- | --- | --- | --- | --- |
|  |  |  |  |  |  |  |  |
|  |  |  |  |  |  |  |  |
| E02 ACA | M23 CTG | 100 | 400 rfu | 19.68 | 0.35 | 4.97 | 39 |
| E07 AGG | M19 CAG | 50 | 5% | 13.11 | 0.67 | 4.89 | 23 |
| E04 ACG | M21 CAT | 100 | 20% | 7.77 | 1.24 | 5.36 | 28 |
| E01 ACT | M21 CTA | 250 | 100 rfu | 9.70 | 0.35 | 5.00 | 10 |
| E02 ACA | M21 CTA | 100 | 250 rfu | 18.74 | 0.21 | 4.41 | 17 |
| E03 AAC | M20 CAT | 150 | 15% | 5.54 | 3.14 | 5.47 | 16 |
| E04 ACC | M17 CAA | 50 | 100 rfu | 19.98 | 0.10 | 5.21 | 30 |
| E05 AGC | M19 CAG | 150 | 15% | 14.47 | 0.62 | 5.00 | 30 |
| E07 AGG | M17 CAA | 150 | 15% | 6.54 | 1.18 | 4.86 | 18 |
| E08 ACG | M22 CTC | 100 | 50 rfu | 13.83 | 0.46 | 5.04 | 31 |
|  |  |  | **Mean** | 12.94 | 0.83 | 5.02 | 24 |
|  |  |  | **Median** | 13.47 | 0.54 | 5.00 | 26 |
|  |  |  | **Sum** | - | - | - | 242 |
