# Supplementary material for: Dealing with AFLP genotyping errors to reveal genetic structure in Plukenetia volubilis (Euphorbiaceae) in the Peruvian Amazon
Source: PLoS One. 2017 Sep 14;12(9):e0184259. doi: 10.1371/journal.pone.0184259 (PMC5598967; doi:10.1371/journal.pone.0184259)
Supplement: S4 Table — The correlation coefficient and p-value for each primer set and concatenated matrix are shown. Statistically significant results are highlighted. (DOCX) [file pone.0184259.s005.docx]

**S4a Table.** Detection of size homoplasy for the “RawGeno” datasets based on a linear regression model describing the correlation between the size of the fragments and their frequency in the population. The correlation coefficient and p-value for each primer set and concatenated matrix are shown. Statistically significant results are highlighted.

| **Primer set** | | **rep-100** | |  | **rep-150** | |  | **all-100** | |  | **all-150** | |
| --- | --- | --- | --- | --- | --- | --- | --- | --- | --- | --- | --- | --- |
|  |  | **r** | **p-value** |  | **r** | **p-value** |  | **r** | **p-value** |  | **r** | **p-value** |
| E02 ACA | M23 CTG | -0.1364 | 0.5777 |  | -0.1112 | 0.6932 |  | 0.0009 | 0.9958 |  | 0.0433 | 0.8267 |
| E07 AGG | M19 CAG | -0.3009 | 0.4690 |  | -0.6243 | 0.1340 |  | -0.1570 | 0.4853 |  | -0.0654 | 0.8030 |
| E04 ACG | M21 CAT | -0.2898 | 0.3368 |  | -0.2898 | 0.3368 |  | -0.1875 | 0.4033 |  | -0.1161 | 0.6464 |
| E01 ACT | M21 CTA | -0.0016 | 0.9944 |  | 0.1109 | 0.7454 |  | -0.0715 | 0.7230 |  | 0.0677 | 0.8180 |
| E02 ACA | M21 CTA | -0.0856 | 0.8266 |  | -0.1358 | 0.7716 |  | -0.2593 | 0.3707 |  | -0.1706 | 0.6607 |
| E03 AAC | M20 CAT | 0.0880 | 0.7550 |  | 0.3249 | 0.2570 |  | 0.1663 | 0.4711 |  | 0.3585 | 0.1576 |
| E04 ACC | M17 CAA | -0.3821 | 0.2202 |  | -0.4723 | 0.1681 |  | -0.1927 | 0.3077 |  | -0.2346 | 0.3337 |
| E05 AGC | M19 CAG | -0.2928 | 0.2541 |  | -0.3576 | 0.1907 |  | -0.2366 | 0.1470 |  | -0.2880 | 0.1298 |
| E07 AGG | M17 CAA | 0.2245 | 0.4609 |  | 0.2245 | 0.4609 |  | 0.0968 | 0.6933 |  | 0.0982 | 0.6983 |
| E08 ACG | M22 CTC | -0.0597 | 0.8026 |  | 0.1333 | 0.6642 |  | -0.0778 | 0.6720 |  | -0.1047 | 0.6792 |
| **Concatenated** | | -0.1497 | 0.0639 |  | -0.1666 | 0.0689 |  | **-0.1324** | **0.0282** |  | -0.1014 | 0.1617 |

**S4b Table.** Detection of size homoplasy for the “Error” datasets based on a linear regression model describing the correlation between the size of the fragments and their frequency in the population. The correlation coefficient and p-value for each primer set and concatenated matrix are shown. Statistically significant results are highlighted.

| **Primer set** | | **error-2** | |  | **error-3** | |  | **error-4** | |  | **error-5** | |
| --- | --- | --- | --- | --- | --- | --- | --- | --- | --- | --- | --- | --- |
|  |  | **r** | **p-value** |  | **r** | **p-value** |  | **r** | **p-value** |  | **r** | **p-value** |
| E02 ACA | M23 CTG | -0.2633 | 0.2620 |  | -0.2633 | 0.262 |  | -0.3026 | 0.1604 |  | -0.2706 | 0.1908 |
| E07 AGG | M19 CAG | 0.4313 | 0.2464 |  | 0.4015 | 0.1738 |  | 0.3760 | 0.2054 |  | 0.2477 | 0.2791 |
| E04 ACG | M21 CAT | 0.2653 | 0.4903 |  | 0.0026 | 0.9951 |  | -0.1977 | 0.4629 |  | -0.1905 | 0.3413 |
| E01 ACT | M21 CTA | -0.0529 | 0.9207 |  | -0.0986 | 0.8526 |  | 0.0749 | 0.9188 |  | -0.0395 | 0.9261 |
| E02 ACA | M21 CTA | 0.2780 | 0.5937 |  | 0.0836 | 0.8936 |  | 0.5910 | 0.0720 |  | -0.1661 | 0.5876 |
| E03 AAC | M20 CAT | -0.2804 | 0.8190 |  | -0.2804 | 0.8190 |  | -0.1683 | 0.7500 |  | 0.0158 | 0.9574 |
| E04 ACC | M17 CAA | 0.0841 | 0.8430 |  | 0.2268 | 0.3102 |  | 0.1763 | 0.3602 |  | 0.0154 | 0.9517 |
| E05 AGC | M19 CAG | 0.2985 | 0.1305 |  | 0.1927 | 0.4567 |  | -0.3462 | 0.3271 |  | 0.2993 | 0.1294 |
| E07 AGG | M17 CAA | -0.7261 | 0.2739 |  | -0.3827 | 0.7500 |  | -0.3463 | 0.3270 |  | -0.0991 | 0.7254 |
| E08 ACG | M22 CTC | -0.0488 | 0.8935 |  | -0.1852 | 0.5857 |  | -0.1296 | 0.6200 |  | -0.1679 | 0.4026 |
| **Concatenated** | | 0.0952 | 0.3412 |  | 0.0416 | 0.6688 |  | 0.0582 | 0.4912 |  | -0.0403 | 0.5770 |
