# Supplementary material for: Dealing with AFLP genotyping errors to reveal genetic structure in Plukenetia volubilis (Euphorbiaceae) in the Peruvian Amazon
Source: PLoS One. 2017 Sep 14;12(9):e0184259. doi: 10.1371/journal.pone.0184259 (PMC5598967; doi:10.1371/journal.pone.0184259)
Supplement: S5 Table — Detection of SH based on a linear regression model with different criteria for the minimum fragment size (150, 200, 250 and 300 bp). The correlation coefficient and p-value for each dataset are shown. Statistically significant results are highlighted. (DOCX) [file pone.0184259.s006.docx]

**S5 Table.** Size homoplasy (SH) decomposition. Detection of SH based on a linear regression model with different criteria for the minimum fragment size (150, 200, 250 and 300 bp). The correlation coefficient and p-value for each dataset are shown. Statistically significant results are highlighted.

| **Dataset** | **150 bp** | |  | **200 bp** | |  | **250 bp** | |  | **300 bp** | |
| --- | --- | --- | --- | --- | --- | --- | --- | --- | --- | --- | --- |
|  | **r** | **p-value** |  | **r** | **p-value** |  | **r** | **p-value** |  | **r** | **p-value** |
| **rep-100** | -0.1867 | 0.0530 |  | -0.2017 | 0.0848 |  | **-0.3255** | **0.0153** |  | -0.3384 | 0.0582 |
| **rep-150** | **-0.2161** | **0.0497** |  | -0.2294 | 0.0833 |  | **-0.3596** | **0.0153** |  | **-0.4487** | **0.0245** |
| **all-100** | -0.1107 | 0.1160 |  | -0.0907 | 0.2814 |  | -0.1530 | 0.1156 |  | -0.1792 | 0.1669 |
| **all-150** | -0.0786 | 0.3593 |  | -0.0918 | 0.3762 |  | -0.2147 | 0.0722 |  | **-0.3569** | **0.0257** |
| **error-2** | 0.0594 | 0.6254 |  | -0.0229 | 0.8672 |  | **-0.3512** | **0.0284** |  | -0.3326 | 0.0968 |
| **error-3** | 0.0282 | 0.8180 |  | -0.0472 | 0.7371 |  | -0.2299 | 0.1481 |  | -0.3348 | 0.1019 |
| **error-4** | 0.1116 | 0.2893 |  | -0.0487 | 0.6824 |  | -0.2177 | 0.1211 |  | **-0.3660** | **0.0467** |
| **error-5** | 0.0385 | 0.6672 |  | -0.0476 | 0.6507 |  | -0.2170 | 0.0826 |  | -0.2418 | 0.1328 |
