# Supplementary material for: Dealing with AFLP genotyping errors to reveal genetic structure in Plukenetia volubilis (Euphorbiaceae) in the Peruvian Amazon
Source: PLoS One. 2017 Sep 14;12(9):e0184259. doi: 10.1371/journal.pone.0184259 (PMC5598967; doi:10.1371/journal.pone.0184259)
Supplement: S6 Table — (DOCX) [file pone.0184259.s007.docx]

**S6 Table.** Comparison of several similarity coefficients for two- and three-dimensional MDS models based on the stress parameter.

| **Dataset** | **2 dimensions** | |  | **3 dimensions** | |
| --- | --- | --- | --- | --- | --- |
|  | **Jaccard/ Dice** | **Simple Match** |  | **Jaccard/ Dice** | **Simple Match** |
| **rep-100** | 0.2309 | 0.2296 |  | 0.1579 | 0.1644 |
| **rep-150** | 0.2146 | 0.2264 |  | 0.1570 | 0.1635 |
| **all-100** | 0.2254 | 0.2391 |  | 0.1706 | 0.1758 |
| **all-150** | 0.2179 | 0.2405 |  | 0.1662 | 0.1751 |
| **error-2** | 0.2339 | 0.2403 |  | 0.1581 | 0.1699 |
| **error-3** | 0.2232 | 0.2307 |  | 0.1654 | 0.1680 |
| **error-4** | 0.2170 | 0.2297 |  | 0.1624 | 0.1675 |
| **error-5** | 0.2184 | 0.2329 |  | 0.1573 | 0.1662 |
