# Supplementary material for: Dealing with AFLP genotyping errors to reveal genetic structure in Plukenetia volubilis (Euphorbiaceae) in the Peruvian Amazon
Source: PLoS One. 2017 Sep 14;12(9):e0184259. doi: 10.1371/journal.pone.0184259 (PMC5598967; doi:10.1371/journal.pone.0184259)
Supplement: S7 Table — The best clustering according to pseudo-F (highest value) and BIC (lowest value) is highlighted. (DOCX) [file pone.0184259.s008.docx]

**S7a Table.** Optimal number of K clusters according to the pseudo-F and Bayesian Information Criterion (BIC) for the “RawGeno” datasets. The best clustering according to pseudo-F (highest value) and BIC (lowest value) is highlighted.

| **K** | **rep-100** |  |  | **rep-150** |  |  | **all-100** |  |  | **all-150** |  |
| --- | --- | --- | --- | --- | --- | --- | --- | --- | --- | --- | --- |
|  | **pseudo-F** | **BIC** |  | **pseudo-F** | **BIC** |  | **pseudo-F** | **BIC** |  | **pseudo-F** | **BIC** |
| 2 | **45.210** | 1669.052 |  | **53.850** | 1564.375 |  | **37.398** | 1754.250 |  | **43.217** | 1640.034 |
| 3 | 37.652 | 1651.911 |  | 41.043 | 1549.290 |  | 29.244 | 1742.761 |  | 31.968 | 1629.253 |
| 4 | 32.822 | 1641.564 |  | 34.212 | 1540.923 |  | 24.574 | 1736.662 |  | 26.383 | 1623.418 |
| 5 | 28.565 | 1636.679 |  | 30.075 | 1535.113 |  | 21.444 | 1733.253 |  | 22.790 | 1620.207 |
| 6 | 25.884 | 1632.627 |  | 26.837 | 1531.930 |  | 19.532 | 1730.282 |  | 20.427 | 1617.948 |
| 7 | 24.010 | 1629.243 |  | 24.493 | 1529.641 |  | 18.404 | 1727.083 |  | 18.716 | 1616.410 |
| 8 | 22.254 | 1627.671 |  | 23.005 | 1526.936 |  | 17.282 | 1725.467 |  | 17.487 | 1615.082 |
| 9 | 20.876 | **1626.524** |  | 21.564 | **1525.760** |  | 16.262 | **1724.836** |  | 16.418 | **1614.580** |

**S7b Table.** Optimal number of K clusters according to the pseudo-F and Bayesian Information Criterion (BIC) for the “Error” datasets. Best clustering according to pseudo-F (highest value) and BIC (lowest value) is highlighted.

| **K** | **error-2** |  |  | **error-3** |  |  | **error-4** |  |  | **error-5** |  |
| --- | --- | --- | --- | --- | --- | --- | --- | --- | --- | --- | --- |
|  | **pseudo-F** | **BIC** |  | **pseudo-F** | **BIC** |  | **pseudo-F** | **BIC** |  | **pseudo-F** | **BIC** |
| 2 | **53.261** | 1540.301 |  | **43.809** | 1557.195 |  | **45.166** | 1632.364 |  | **38.082** | 1731.961 |
| 3 | 45.724 | 1518.725 |  | 40.034 | 1535.757 |  | 38.787 | 1613.655 |  | 31.672 | 1717.503 |
| 4 | 38.521 | 1508.744 |  | 34.355 | 1525.792 |  | 32.371 | 1605.680 |  | 26.197 | 1711.620 |
| 5 | 33.626 | 1502.705 |  | 31.483 | 1517.065 |  | 29.589 | 1597.587 |  | 23.484 | 1706.268 |
| 6 | 30.498 | 1497.766 |  | 28.989 | 1511.314 |  | 26.811 | 1593.361 |  | 21.360 | 1702.937 |
| 7 | 28.486 | 1493.097 |  | 26.915 | 1507.362 |  | 25.110 | 1589.080 |  | 19.787 | 1700.475 |
| 8 | 26.656 | 1490.150 |  | 24.896 | 1505.589 |  | 23.319 | 1587.204 |  | 18.289 | 1699.731 |
| 9 | 25.213 | **1487.746** |  | 23.467 | **1503.700** |  | 22.037 | **1585.301** |  | 17.225 | **1698.863** |
