# Supplementary material for: Dealing with AFLP genotyping errors to reveal genetic structure in Plukenetia volubilis (Euphorbiaceae) in the Peruvian Amazon
Source: PLoS One. 2017 Sep 14;12(9):e0184259. doi: 10.1371/journal.pone.0184259 (PMC5598967; doi:10.1371/journal.pone.0184259)
Supplement: S8 Table — (DOCX) [file pone.0184259.s009.docx]

**S8a Table. Pairwise population Φ_PT_ values.** Φ_PT_ values are shown below diagonal and p-values based on 9999 permutation are shown above diagonal.

| **rep-100** | **2DM** | **ADO** | **AUC** | **CHU** | **MIS** | **PAC** | **PUC** | **RAC** | **SCR** |
| --- | --- | --- | --- | --- | --- | --- | --- | --- | --- |
| **2DM** |  | 0.000 | 0.000 | 0.000 | 0.000 | 0.000 | 0.000 | 0.000 | 0.000 |
| **ADO** | 0.536 |  | 0.000 | 0.000 | 0.000 | 0.000 | 0.000 | 0.000 | 0.000 |
| **AUC** | 0.426 | 0.315 |  | 0.000 | 0.000 | 0.000 | 0.000 | 0.000 | 0.000 |
| **CHU** | 0.453 | 0.208 | 0.177 |  | 0.000 | 0.000 | 0.000 | 0.000 | 0.000 |
| **MIS** | 0.572 | 0.354 | 0.364 | 0.223 |  | 0.000 | 0.000 | 0.000 | 0.000 |
| **PAC** | 0.538 | 0.270 | 0.295 | 0.157 | 0.160 |  | 0.000 | 0.000 | 0.000 |
| **PUC** | 0.489 | 0.297 | 0.191 | 0.125 | 0.204 | 0.122 |  | 0.000 | 0.001 |
| **RAC** | 0.527 | 0.405 | 0.298 | 0.219 | 0.449 | 0.390 | 0.331 |  | 0.000 |
| **SCR** | 0.435 | 0.250 | 0.155 | 0.085 | 0.151 | 0.068 | 0.044 | 0.215 |  |

**S8b Table. Pairwise population Φ_PT_ values.** Φ_PT_ values are shown below diagonal and p-values based on 9999 permutation are shown above diagonal.

| **rep-150** | **2DM** | **ADO** | **AUC** | **CHU** | **MIS** | **PAC** | **PUC** | **RAC** | **SCR** |
| --- | --- | --- | --- | --- | --- | --- | --- | --- | --- |
| **2DM** |  | 0.000 | 0.000 | 0.000 | 0.000 | 0.000 | 0.000 | 0.000 | 0.000 |
| **ADO** | 0.542 |  | 0.000 | 0.000 | 0.000 | 0.000 | 0.000 | 0.000 | 0.000 |
| **AUC** | 0.420 | 0.296 |  | 0.000 | 0.000 | 0.000 | 0.000 | 0.000 | 0.000 |
| **CHU** | 0.473 | 0.156 | 0.191 |  | 0.000 | 0.000 | 0.000 | 0.000 | 0.000 |
| **MIS** | 0.574 | 0.274 | 0.355 | 0.251 |  | 0.000 | 0.000 | 0.000 | 0.000 |
| **PAC** | 0.552 | 0.175 | 0.257 | 0.150 | 0.184 |  | 0.000 | 0.000 | 0.000 |
| **PUC** | 0.486 | 0.218 | 0.155 | 0.143 | 0.228 | 0.127 |  | 0.000 | 0.000 |
| **RAC** | 0.535 | 0.390 | 0.281 | 0.180 | 0.443 | 0.363 | 0.269 |  | 0.000 |
| **SCR** | 0.440 | 0.200 | 0.127 | 0.099 | 0.193 | 0.081 | 0.049 | 0.173 |  |

**S8c Table. Pairwise population Φ_PT_ values.** Φ_PT_ values are shown below diagonal and p-values based on 9999 permutation are shown above diagonal.

| **all-100** | **2DM** | **ADO** | **AUC** | **CHU** | **MIS** | **PAC** | **PUC** | **RAC** | **SCR** |
| --- | --- | --- | --- | --- | --- | --- | --- | --- | --- |
| **2DM** |  | 0.000 | 0.000 | 0.000 | 0.000 | 0.000 | 0.000 | 0.000 | 0.000 |
| **ADO** | 0.452 |  | 0.000 | 0.000 | 0.000 | 0.000 | 0.000 | 0.000 | 0.000 |
| **AUC** | 0.379 | 0.269 |  | 0.000 | 0.000 | 0.000 | 0.000 | 0.000 | 0.000 |
| **CHU** | 0.391 | 0.165 | 0.154 |  | 0.000 | 0.000 | 0.000 | 0.000 | 0.000 |
| **MIS** | 0.479 | 0.281 | 0.310 | 0.178 |  | 0.000 | 0.000 | 0.000 | 0.000 |
| **PAC** | 0.456 | 0.217 | 0.256 | 0.132 | 0.121 |  | 0.000 | 0.000 | 0.000 |
| **PUC** | 0.419 | 0.242 | 0.167 | 0.105 | 0.158 | 0.100 |  | 0.000 | 0.002 |
| **RAC** | 0.436 | 0.323 | 0.266 | 0.169 | 0.350 | 0.303 | 0.270 |  | 0.000 |
| **SCR** | 0.371 | 0.199 | 0.135 | 0.073 | 0.123 | 0.063 | 0.036 | 0.167 |  |

**S8d Table. Pairwise population Φ_PT_ values.** Φ_PT_ values are shown below diagonal and p-values based on 9999 permutation are shown above diagonal.

| **all-150** | **2DM** | **ADO** | **AUC** | **CHU** | **MIS** | **PAC** | **PUC** | **RAC** | **SCR** |
| --- | --- | --- | --- | --- | --- | --- | --- | --- | --- |
| **2DM** |  | 0.000 | 0.000 | 0.000 | 0.000 | 0.000 | 0.000 | 0.000 | 0.000 |
| **ADO** | 0.484 |  | 0.000 | 0.000 | 0.000 | 0.000 | 0.000 | 0.000 | 0.000 |
| **AUC** | 0.379 | 0.259 |  | 0.000 | 0.000 | 0.000 | 0.000 | 0.000 | 0.000 |
| **CHU** | 0.422 | 0.130 | 0.168 |  | 0.000 | 0.000 | 0.000 | 0.000 | 0.000 |
| **MIS** | 0.493 | 0.223 | 0.303 | 0.205 |  | 0.000 | 0.000 | 0.000 | 0.000 |
| **PAC** | 0.481 | 0.140 | 0.218 | 0.121 | 0.131 |  | 0.000 | 0.000 | 0.000 |
| **PUC** | 0.424 | 0.183 | 0.136 | 0.120 | 0.173 | 0.092 |  | 0.000 | 0.001 |
| **RAC** | 0.467 | 0.331 | 0.249 | 0.143 | 0.347 | 0.276 | 0.214 |  | 0.000 |
| **SCR** | 0.386 | 0.164 | 0.112 | 0.087 | 0.152 | 0.065 | 0.036 | 0.135 |  |

**S8e Table. Pairwise population Φ_PT_ values.** Φ_PT_ values are shown below diagonal and p-values based on 9999 permutation are shown above diagonal.

| **error-2** | **2DM** | **ADO** | **AUC** | **CHU** | **MIS** | **PAC** | **PUC** | **RAC** | **SCR** |
| --- | --- | --- | --- | --- | --- | --- | --- | --- | --- |
| **2DM** |  | 0.000 | 0.000 | 0.000 | 0.000 | 0.000 | 0.000 | 0.000 | 0.000 |
| **ADO** | 0.641 |  | 0.000 | 0.000 | 0.000 | 0.000 | 0.000 | 0.000 | 0.000 |
| **AUC** | 0.485 | 0.291 |  | 0.000 | 0.000 | 0.000 | 0.000 | 0.000 | 0.000 |
| **CHU** | 0.527 | 0.153 | 0.137 |  | 0.000 | 0.000 | 0.000 | 0.000 | 0.000 |
| **MIS** | 0.635 | 0.294 | 0.392 | 0.286 |  | 0.000 | 0.000 | 0.000 | 0.000 |
| **PAC** | 0.617 | 0.138 | 0.263 | 0.178 | 0.177 |  | 0.000 | 0.000 | 0.001 |
| **PUC** | 0.567 | 0.174 | 0.170 | 0.117 | 0.245 | 0.107 |  | 0.000 | 0.017 |
| **RAC** | 0.635 | 0.438 | 0.297 | 0.204 | 0.491 | 0.420 | 0.317 |  | 0.000 |
| **SCR** | 0.498 | 0.157 | 0.142 | 0.100 | 0.227 | 0.072 | 0.031 | 0.219 |  |

**S8f Table. Pairwise population Φ_PT_ values.** Φ_PT_ values are shown below diagonal and p-values based on 9999 permutation are shown above diagonal.

| **error-3** | **2DM** | **ADO** | **AUC** | **CHU** | **MIS** | **PAC** | **PUC** | **RAC** | **SCR** |
| --- | --- | --- | --- | --- | --- | --- | --- | --- | --- |
| **2DM** |  | 0.000 | 0.000 | 0.000 | 0.000 | 0.000 | 0.000 | 0.000 | 0.000 |
| **ADO** | 0.630 |  | 0.000 | 0.000 | 0.000 | 0.000 | 0.000 | 0.000 | 0.000 |
| **AUC** | 0.501 | 0.329 |  | 0.000 | 0.000 | 0.000 | 0.000 | 0.000 | 0.000 |
| **CHU** | 0.518 | 0.159 | 0.179 |  | 0.000 | 0.000 | 0.000 | 0.000 | 0.000 |
| **MIS** | 0.607 | 0.301 | 0.411 | 0.280 |  | 0.000 | 0.000 | 0.000 | 0.000 |
| **PAC** | 0.594 | 0.146 | 0.284 | 0.175 | 0.171 |  | 0.000 | 0.000 | 0.001 |
| **PUC** | 0.550 | 0.188 | 0.185 | 0.125 | 0.234 | 0.090 |  | 0.000 | 0.013 |
| **RAC** | 0.631 | 0.414 | 0.315 | 0.184 | 0.423 | 0.359 | 0.268 |  | 0.000 |
| **SCR** | 0.478 | 0.181 | 0.158 | 0.119 | 0.219 | 0.072 | 0.033 | 0.171 |  |

**S8g Table. Pairwise population Φ_PT_ values.** Φ_PT_ values are shown below diagonal and p-values based on 9999 permutation are shown above diagonal.

| **error-4** | **2DM** | **ADO** | **AUC** | **CHU** | **MIS** | **PAC** | **PUC** | **RAC** | **SCR** |
| --- | --- | --- | --- | --- | --- | --- | --- | --- | --- |
| **2DM** |  | 0.000 | 0.000 | 0.000 | 0.000 | 0.000 | 0.000 | 0.000 | 0.000 |
| **ADO** | 0.571 |  | 0.000 | 0.000 | 0.000 | 0.000 | 0.000 | 0.000 | 0.000 |
| **AUC** | 0.472 | 0.316 |  | 0.000 | 0.000 | 0.000 | 0.000 | 0.000 | 0.000 |
| **CHU** | 0.477 | 0.156 | 0.176 |  | 0.000 | 0.000 | 0.000 | 0.000 | 0.000 |
| **MIS** | 0.601 | 0.298 | 0.410 | 0.275 |  | 0.000 | 0.000 | 0.000 | 0.000 |
| **PAC** | 0.557 | 0.173 | 0.298 | 0.157 | 0.170 |  | 0.000 | 0.000 | 0.000 |
| **PUC** | 0.516 | 0.210 | 0.184 | 0.113 | 0.236 | 0.097 |  | 0.000 | 0.001 |
| **RAC** | 0.600 | 0.405 | 0.312 | 0.200 | 0.437 | 0.380 | 0.269 |  | 0.000 |
| **SCR** | 0.455 | 0.202 | 0.159 | 0.115 | 0.223 | 0.082 | 0.047 | 0.192 |  |

**S8h Table. Pairwise population Φ_PT_ values.** Φ_PT_ values are shown below diagonal and p-values based on 9999 permutation are shown above diagonal.

| **error-5** | **2DM** | **ADO** | **AUC** | **CHU** | **MIS** | **PAC** | **PUC** | **RAC** | **SCR** |
| --- | --- | --- | --- | --- | --- | --- | --- | --- | --- |
| **2DM** |  | 0.000 | 0.000 | 0.000 | 0.000 | 0.000 | 0.000 | 0.000 | 0.000 |
| **ADO** | 0.452 |  | 0.000 | 0.000 | 0.000 | 0.000 | 0.000 | 0.000 | 0.000 |
| **AUC** | 0.357 | 0.238 |  | 0.000 | 0.000 | 0.000 | 0.000 | 0.000 | 0.000 |
| **CHU** | 0.406 | 0.130 | 0.147 |  | 0.000 | 0.000 | 0.000 | 0.000 | 0.000 |
| **MIS** | 0.516 | 0.237 | 0.345 | 0.248 |  | 0.000 | 0.000 | 0.000 | 0.000 |
| **PAC** | 0.480 | 0.139 | 0.262 | 0.162 | 0.160 |  | 0.000 | 0.000 | 0.000 |
| **PUC** | 0.432 | 0.169 | 0.150 | 0.095 | 0.201 | 0.117 |  | 0.000 | 0.010 |
| **RAC** | 0.458 | 0.274 | 0.211 | 0.177 | 0.413 | 0.343 | 0.233 |  | 0.000 |
| **SCR** | 0.377 | 0.146 | 0.125 | 0.088 | 0.202 | 0.087 | 0.031 | 0.167 |  |
