# Supplementary material for: Dealing with AFLP genotyping errors to reveal genetic structure in Plukenetia volubilis (Euphorbiaceae) in the Peruvian Amazon
Source: PLoS One. 2017 Sep 14;12(9):e0184259. doi: 10.1371/journal.pone.0184259 (PMC5598967; doi:10.1371/journal.pone.0184259)
Supplement: S9 Table — (DOCX) [file pone.0184259.s010.docx]

**S9** **Table.** Genetic diversity (He) of each subpopulation.

| **Locality** | **Dataset** | | | | | | | |
| --- | --- | --- | --- | --- | --- | --- | --- | --- |
|  | **rep-100** | **rep-150** | **all-100** | **all-150** | **error-2** | **error-3** | **error-4** | **error-5** |
| **2DM** | 0.150 | 0.156 | 0.110 | 0.118 | 0.092 | 0.086 | 0.112 | 0.143 |
| **ADO** | 0.157 | 0.174 | 0.105 | 0.122 | 0.146 | 0.136 | 0.156 | 0.173 |
| **AUC** | 0.215 | 0.236 | 0.128 | 0.156 | 0.183 | 0.169 | 0.171 | 0.200 |
| **CHU** | 0.225 | 0.219 | 0.145 | 0.151 | 0.215 | 0.206 | 0.211 | 0.204 |
| **MIS** | 0.188 | 0.179 | 0.134 | 0.139 | 0.153 | 0.166 | 0.174 | 0.170 |
| **PAC** | 0.159 | 0.139 | 0.115 | 0.110 | 0.142 | 0.147 | 0.155 | 0.150 |
| **PUC** | 0.212 | 0.220 | 0.138 | 0.161 | 0.179 | 0.183 | 0.190 | 0.185 |
| **RAC** | 0.084 | 0.077 | 0.058 | 0.061 | 0.079 | 0.083 | 0.064 | 0.081 |
| **SCR** | 0.249 | 0.247 | 0.163 | 0.178 | 0.215 | 0.225 | 0.223 | 0.206 |
| **Mean** | 0.188 | 0.179 | 0.128 | 0.139 | 0.153 | 0.166 | 0.171 | 0.173 |
