# Supplementary material for: Dealing with AFLP genotyping errors to reveal genetic structure in Plukenetia volubilis (Euphorbiaceae) in the Peruvian Amazon
Source: PLoS One. 2017 Sep 14;12(9):e0184259. doi: 10.1371/journal.pone.0184259 (PMC5598967; doi:10.1371/journal.pone.0184259)
Supplement: S10 Table — The correlation coefficient and p-value are shown. (DOCX) [file pone.0184259.s011.docx]

**S10 Table.** Spatial analysis to test the statistical relationship between genetic and geographical distance by the Mantel test with 9999 random permutations. The correlation coefficient and p-value are shown.

| **Dataset** |  | **r** |  | **p-value** |
| --- | --- | --- | --- | --- |
| **rep-100** |  | 0.474 |  | 0.086 |
| **rep-150** |  | 0.461 |  | 0.093 |
| **all-100** |  | 0.461 |  | 0.092 |
| **all-150** |  | 0.457 |  | 0.106 |
| **error-2** |  | 0.501 |  | 0.061 |
| **error-3** |  | 0.449 |  | 0.105 |
| **error-4** |  | 0.464 |  | 0.096 |
| **error-5** |  | 0.427 |  | 0.099 |
