# Supplementary material for: Dealing with AFLP genotyping errors to reveal genetic structure in Plukenetia volubilis (Euphorbiaceae) in the Peruvian Amazon
Source: PLoS One. 2017 Sep 14;12(9):e0184259. doi: 10.1371/journal.pone.0184259 (PMC5598967; doi:10.1371/journal.pone.0184259)
Supplement: S11 Table — (DOCX) [file pone.0184259.s012.docx]

**S11 Table.** Spatial analysis in the frame of sPCA where the presence of a global and/or local spatial pattern was tested by the G and L test.

| **Dataset** | **Gtest** |  |  | **Ltest** |  |
| --- | --- | --- | --- | --- | --- |
|  | **t(max)** | **p-value** |  | **t(max)** | **p-value** |
| **rep-100** | 0.1899 | 0.2891 |  | 0.1999 | 0.5789 |
| **rep-150** | 0.2107 | 0.2343 |  | 0.2192 | 0.5528 |
| **all-100** | 0.1604 | 0.3511 |  | 0.1650 | 0.6173 |
| **all-150** | 0.1824 | 0.2079 |  | 0.1708 | 0.7035 |
| **error-2** | 0.1934 | 0.1003 |  | 0.1668 | 0.6767 |
| **error-3** | 0.1870 | 0.1269 |  | 0.1621 | 0.7312 |
| **error-4** | 0.1835 | 0.2036 |  | 0.1629 | 0.7868 |
| **error-5** | 0.1773 | 0.2370 |  | 0.1645 | 0.7531 |
