# Supplementary material for: Dealing with AFLP genotyping errors to reveal genetic structure in Plukenetia volubilis (Euphorbiaceae) in the Peruvian Amazon
Source: PLoS One. 2017 Sep 14;12(9):e0184259. doi: 10.1371/journal.pone.0184259 (PMC5598967; doi:10.1371/journal.pone.0184259)
Supplement: S12 Table — The genetic distance matrix was based on ΦPT values. Statistically significant results are highlighted. (DOCX) [file pone.0184259.s013.docx]

**S12 Table.** Numerical results of spatial autocorrelation analysis using the random permutation procedure for seven equidistant classes (10 km). The genetic distance matrix was based on Φ_PT_ values. Statistically significant results are highlighted.

| **Dataset** | **Distance 10 km** | |  | **Distance 20 km** | |  | **Distance 30 km** | |  | **Distance 40 km** | |  | **Distance 50 km** | |  | **Distance 60 km** | |  | **Distance 70 km** | |
| --- | --- | --- | --- | --- | --- | --- | --- | --- | --- | --- | --- | --- | --- | --- | --- | --- | --- | --- | --- | --- |
|  | **r** | **p-value** |  | **r** | **p-value** |  | **r** | **p-value** |  | **r** | **p-value** |  | **r** | **p-value** |  | **r** | **p-value** |  | **r** | **p-value** |
| **rep-100** | -0.006 | 0.506 |  | 0.073 | 0.104 |  | 0.131 | 0.135 |  | 0.053 | 0.222 |  | **-0.103** | **0.034** |  | -0.022 | 0.366 |  | 0.018 | 0.450 |
| **rep-150** | -0.057 | 0.274 |  | **0.112** | **0.044** |  | 0.154 | 0.115 |  | 0.011 | 0.470 |  | -0.085 | 0.073 |  | -0.018 | 0.382 |  | -0.019 | 0.452 |
| **all-100** | -0.001 | 0.546 |  | 0.076 | 0.104 |  | 0.134 | 0.139 |  | 0.027 | 0.350 |  | **-0.099** | **0.047** |  | -0.016 | 0.408 |  | 0.026 | 0.441 |
| **all-150** | -0.060 | 0.254 |  | **0.118** | **0.041** |  | 0.159 | 0.117 |  | -0.001 | 0.470 |  | -0.079 | 0.078 |  | -0.017 | 0.386 |  | -0.035 | 0.387 |
| **error-2** | -0.070 | 0.240 |  | 0.132 | 0.052 |  | 0.238 | 0.053 |  | 0.047 | 0.298 |  | -0.092 | 0.071 |  | -0.037 | 0.298 |  | -0.068 | 0.287 |
| **error-3** | -0.084 | 0.182 |  | 0.118 | 0.056 |  | 0.198 | 0.074 |  | 0.010 | 0.466 |  | -0.074 | 0.103 |  | -0.032 | 0.315 |  | -0.029 | 0.404 |
| **error-4** | -0.086 | 0.137 |  | **0.118** | **0.037** |  | 0.164 | 0.094 |  | 0.015 | 0.440 |  | -0.081 | 0.074 |  | -0.023 | 0.363 |  | -0.016 | 0.445 |
| **error-5** | -0.048 | 0.332 |  | 0.098 | 0.056 |  | 0.198 | 0.077 |  | 0.052 | 0.271 |  | **-0.095** | **0.047** |  | -0.047 | 0.255 |  | 0.040 | 0.446 |
