# Supplementary figures and images for: Dealing with AFLP genotyping errors to reveal genetic structure in Plukenetia volubilis (Euphorbiaceae) in the Peruvian Amazon
Source: PLoS One. 2017 Sep 14;12(9):e0184259. doi: 10.1371/journal.pone.0184259 (PMC5598967; doi:10.1371/journal.pone.0184259)

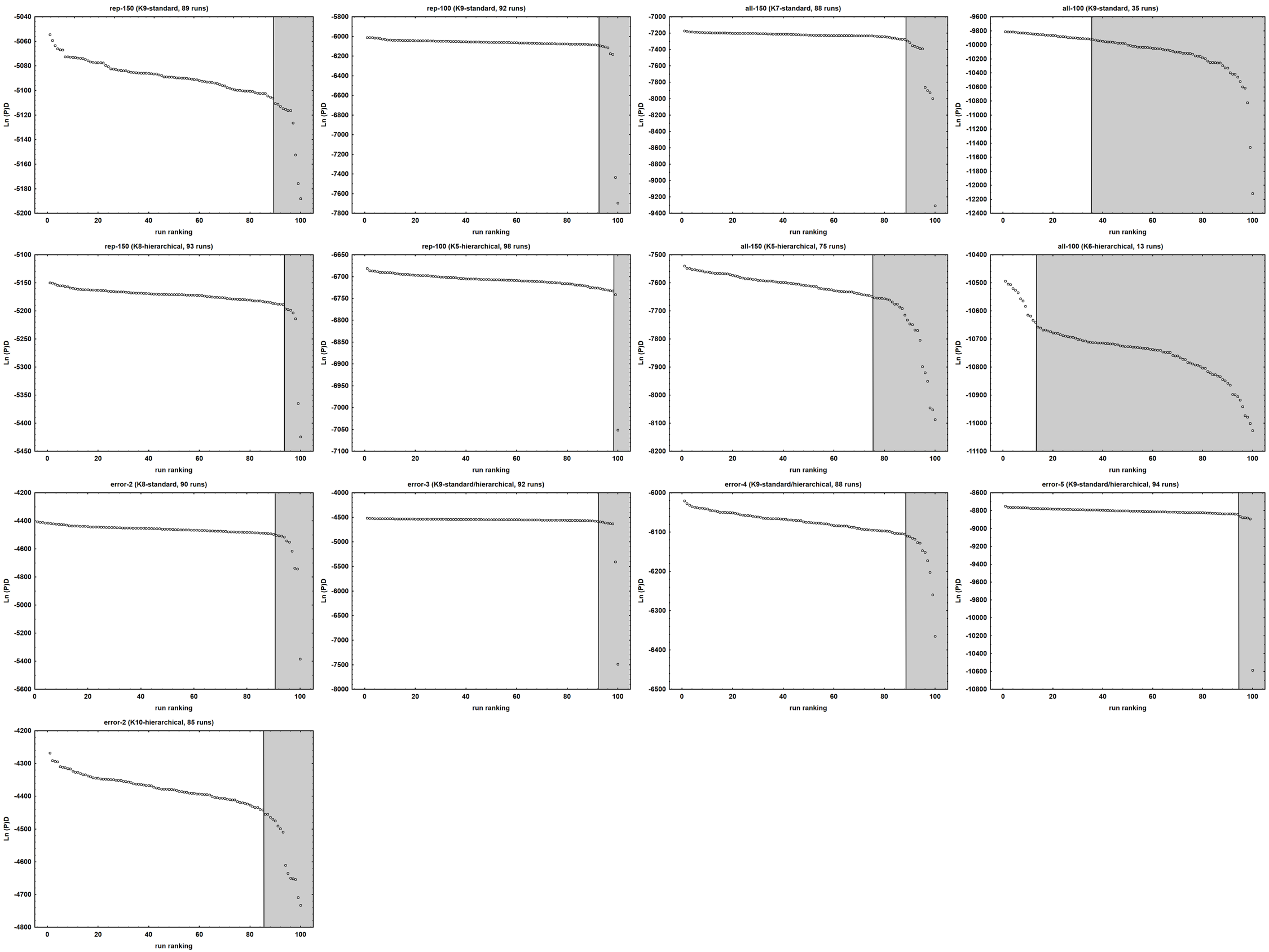

Supplement: S1 Fig — The number of retained runs is written in brackets together with the K value and the type of STRUCTURE analysis (“standard” and/or “hierarchical”). Please note the different scales of the Ln(P)D axes. (TIF) [file pone.0184259.s014.tif]

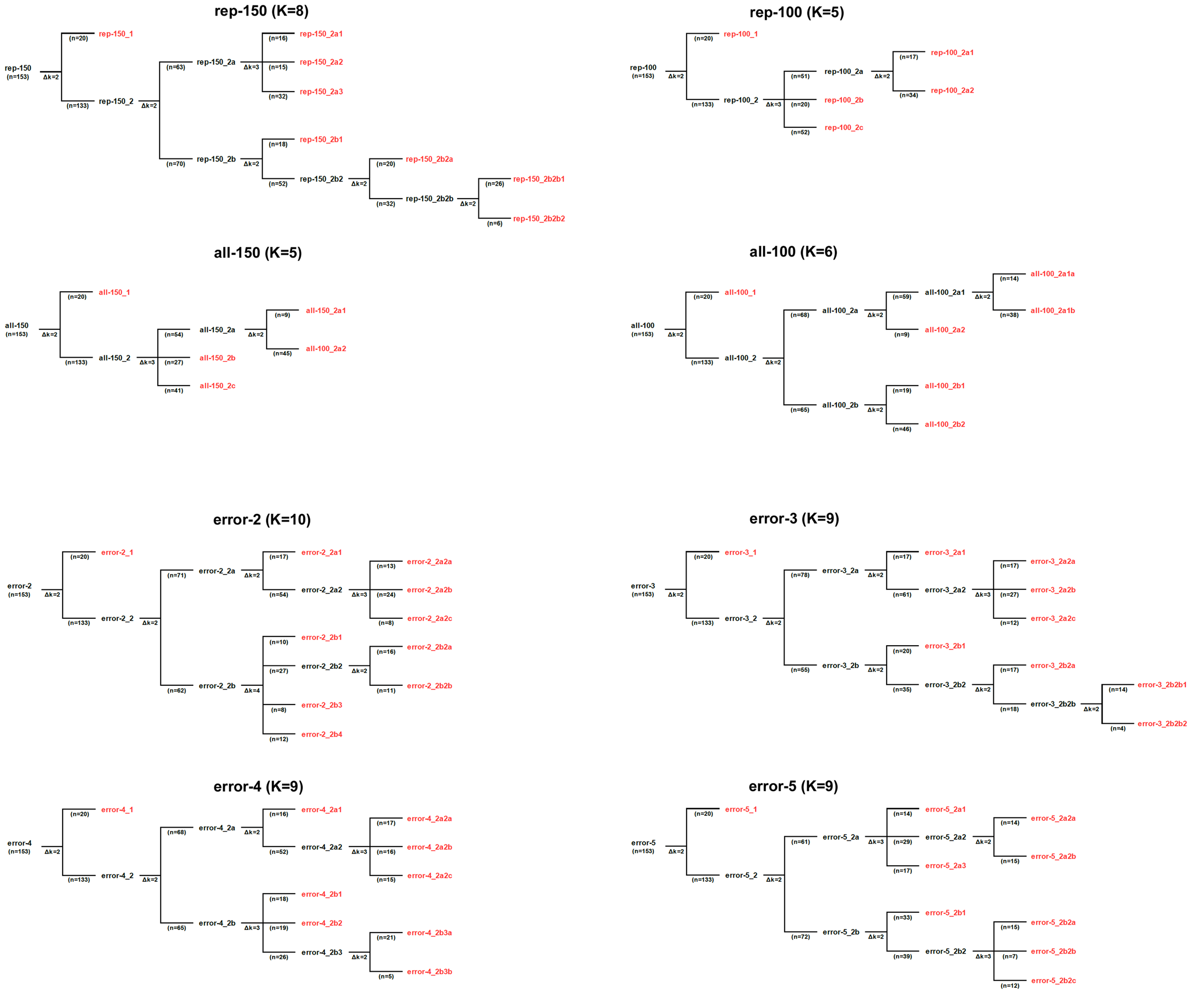

Supplement: S2 Fig — Estimation of the K number according to ΔK for each round of hierarchical analysis and the number of individuals within the cluster is shown. Red colored clusters represent clusters which could not be divided any further. (TIF) [file pone.0184259.s015.tif]

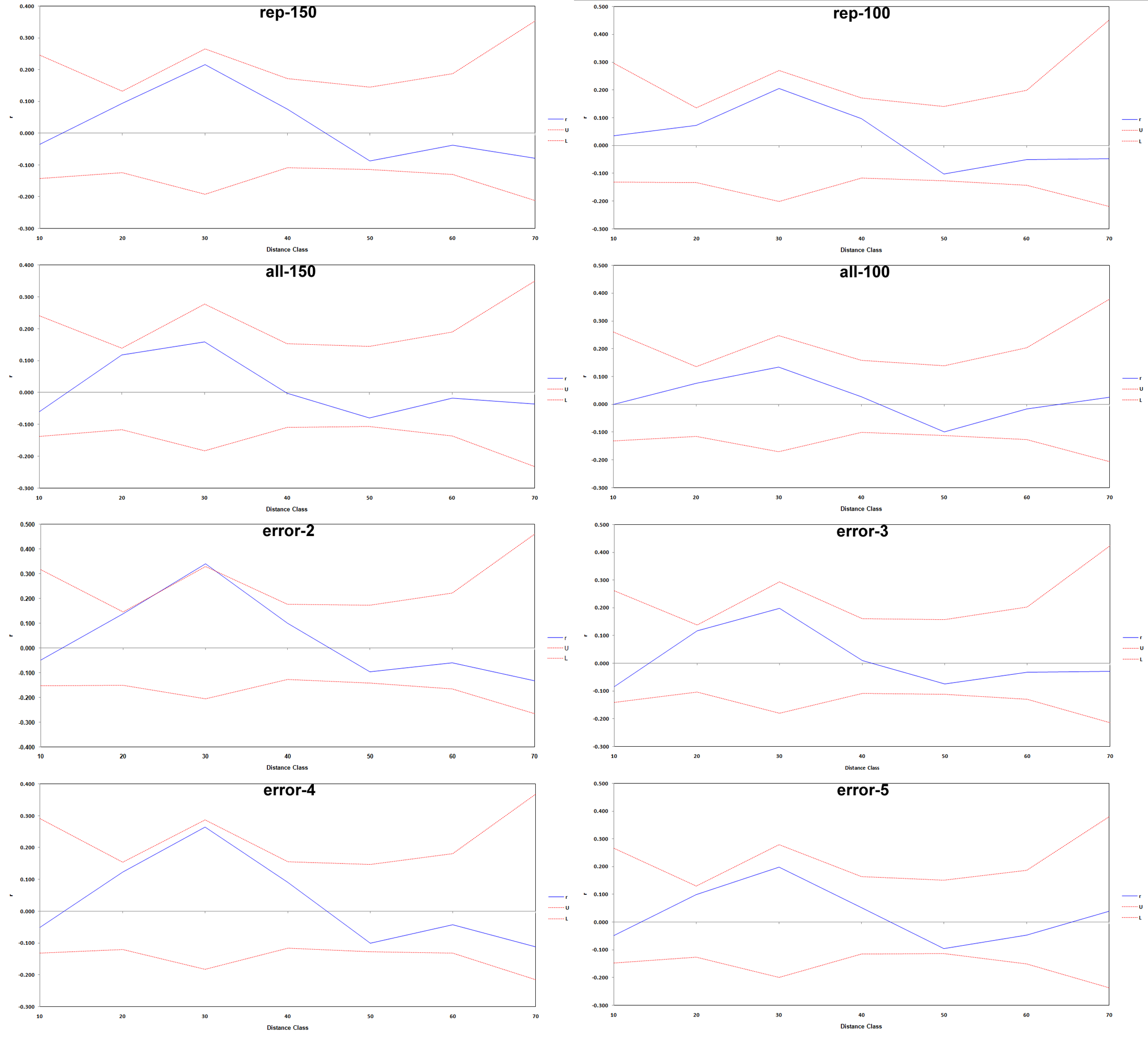

Supplement: S3 Fig — The calculated r value (blue line) is shown with upper (U) and lower (L) bounds of the 95% confidence interval (red lines). (TIF) [file pone.0184259.s016.tif]
